# ABHD5/CGI-58, the Chanarin-Dorfman Syndrome Protein, Mobilises Lipid Stores for Hepatitis C Virus Production

**DOI:** 10.1371/journal.ppat.1005568

**Published:** 2016-04-28

**Authors:** Gabrielle Vieyres, Kathrin Welsch, Gisa Gerold, Juliane Gentzsch, Sina Kahl, Florian W. R. Vondran, Lars Kaderali, Thomas Pietschmann

**Affiliations:** 1 Institute of Experimental Virology, TWINCORE, Centre for Experimental and Clinical Infection Research; a joint venture between the Medical School Hannover (MHH) and the Helmholtz Centre for Infection Research (HZI), Hannover, Germany; 2 German Centre for Infection Research (DZIF), partner site Hannover-Braunschweig, Braunschweig, Germany; 3 ReMediES, Department of General, Visceral and Transplant Surgery, Hannover Medical School, Hannover, Germany; 4 Institute for Bioinformatics, University Medicine Greifswald, Greifswald, Germany; The Scripps Research Institute, UNITED STATES

## Abstract

Hepatitis C virus (HCV) particles closely mimic human very-low-density lipoproteins (VLDL) to evade humoral immunity and to facilitate cell entry. However, the principles that govern HCV association with VLDL components are poorly defined. Using an siRNA screen, we identified ABHD5 (α/β hydrolase domain containing protein 5, also known as CGI-58) as a new host factor promoting both virus assembly and release. ABHD5 associated with lipid droplets and triggered their hydrolysis. Importantly, ABHD5 Chanarin-Dorfman syndrome mutants responsible for a rare lipid storage disorder in humans were mislocalised, and unable to consume lipid droplets or support HCV production. Additional ABHD5 mutagenesis revealed a novel tribasic motif that does not influence subcellular localization but determines both ABHD5 lipolytic and proviral properties. These results indicate that HCV taps into the lipid droplet triglyceride reservoir usurping ABHD5 lipase cofactor function. They also suggest that the resulting lipid flux, normally devoted to VLDL synthesis, also participates in the assembly and release of the HCV lipo-viro-particle. Altogether, our study provides the first association between the Chanarin-Dorfman syndrome protein and an infectious disease and sheds light on the hepatic manifestations of this rare genetic disorder as well as on HCV morphogenesis.

## Author Summary

HCV replication is linked to the host lipid metabolism, and virions are secreted as lipo-viro-particles whose density, size and biochemical content resemble VLDL. HCV assembles close to lipid droplets and is released via the secretory pathway, but it remains unclear how it accesses the VLDL assembly pathway. In this study, we identified ABHD5 as a new host factor supporting HCV assembly and release. ABHD5 is a lipid droplet-associated lipase cofactor. In hepatocytes, ABHD5 was proposed to promote the recruitment of triglycerides from cytosolic towards luminal lipid droplets by mediating a cycle of phospholipid hydrolysis/re-esterification. Our data suggest that this ABHD5-dependent lipid transfer is not only required for VLDL maturation, but also for HCV assembly and virion release, indicating that lipid remodelling impacts on both assembly and virus transport. Finally, ABHD5 is associated with the Chanarin-Dorfman syndrome, a rare human genetic lipid metabolism disorder. We found that the Chanarin-Dorfman syndrome mutants were unable to support HCV assembly, pointing at a new host polymorphism that could determine susceptibility to HCV infection. Altogether, our results establish a new link between HCV, VLDL assembly and lipid remodeling pathways and open new possibilities to study the etiology of the liver manifestations of the Chanarin-Dorfman syndrome.

## Introduction

HCV chronically infects around 146 million people worldwide [1] and the associated cases of end-stage liver disease constitute a major indication for liver transplantation [2]. A hallmark of chronic hepatitis C is the dysregulation of the host lipid metabolism, notably with the occurrence of liver steatosis in 40% of chronically infected patients in absence of any other predisposition factor [3]. Curiously, the virion strikingly resembles very low density lipoproteins (VLDL) with its unusually low buoyant density, its association with apolipoproteins and peculiar lipid content [4–8]. This mimicry enables the use of lipid receptors in the virus entry process and facilitates the homing to liver cells as well as antibody escape [9]. It also suggested the involvement of the VLDL synthesis pathway in HCV morphogenesis. Indeed, in the course of virus assembly HCV modulates lipid droplets (LD), the principal cellular lipid storage organelles, by deposition of viral components and manipulation of LD motility [10,11]. This process is probably initiated by the viral core protein, which uses DGAT1 to translocate from the ER membrane onto the lipid droplet (LD) surface [12]. Assembly complexes are then built in the vicinity of the replication complexes and straddle ER membrane and lipid droplets [10]. Importantly, the VLDL-associated apolipoprotein ApoE is a critical factor for HCV assembly, while other liver-specific components of the VLDL machinery might be involved but can be bypassed [13,14]. However, the mechanisms that mediate the loading of HCV particles with apolipoproteins and VLDL lipids are poorly understood.

ABHD5 is the causative gene for the Chanarin-Dorfman syndrome (CDS), a neutral lipid storage disorder associated with ichtyosis [15]. This very rare autosomal recessive disease affects multiple organs including the liver, with frequent cases of steatosis or hepatomegaly [16]. Despite its homology with other lipid hydrolases, ABHD5 lacks a direct lipase activity. However, two functions have been attributed to the protein: (i) a putative lysophosphatidic acid acyltransferase (LPAAT) activity, and (ii) a demonstrated lipase cofactor activity [15]. ABHD5 binds to lipid droplets and promotes the triglyceride mobilisation from their LD storage by activating hydrolysis in response to lipolytic stimulation [15]. Although mainly studied in adipocytes, ABHD5 expression and lipase cofactor activity seem to be ubiquitous. The fate of the mobilised lipids however depends on the tissue [17]. In hepatocytes, they may engage in the VLDL synthesis pathway: according to the current model, the hydrolyzed lipids are re-esterified at the ER membrane, forming luminal LDs that fuse with ApoB-positive VLDL precursors before secretion [18–20].

Using a rational siRNA screen, we identified ABHD5 as a new host factor participating in HCV morphogenesis. Moreover, we report that mutants of this lipase cofactor, responsible for the Chanarin-Dorfman syndrome, were unable to support virus production. We further expand on the molecular biology of ABHD5 with the description of a new tribasic motif that specifically mediates its lipid droplet consumption function. Finally, our data specifically link ABHD5 ability to trigger a lipid flux with its proviral effect on both HCV assembly and release.

## Results

### An siRNA-based screen identifies ABHD5 as novel HCV assembly/release host dependency factor

To identify novel host factors involved in the loading of lipoproteins onto HCV particles and given the close relationship between the HCV replication cycle and host lipid metabolism we specifically screened host genes that have previously been implicated in modulating lipid droplet function/morphology and lipoprotein secretion. First of all, a fraction of our candidates were selected from published genome-wide siRNA screens identifying host factors involved in lipid droplet formation, maintenance and dynamics [21,22]. Among the extensive lists of genes reported by these authors, we eliminated housekeeping genes and shortlisted 11 genes (PSCM3, UBE2D3, DYNLRB1, YWHAE, PLA2G6, ARF1, PCYT1A, CHKA, FASN, SEC22B and BSCL2) representing all the different lipid droplet phenotypes observed by these authors upon gene knockdown. A second category of candidates (RAB18, CES1, CES3, ABHD5, FABP1, PLD1, PLIN2 (ADRP), PLIN3 (TIP47)) was selected upon their specific functional involvement in host lipid homeostasis or lipoprotein metabolism [23–27]. Among these, FABP1 is an abundant cytosolic lipid chaperone transporting fatty acids to diverse cell organelles, including the lipid droplets [26]. PLD1 has been involved in the formation of lipid droplets [28] and, in collaboration with ARF1, of VLDL [29]. ADRP and TIP47 belong to the lipid droplet-associated PAT protein family (perilipin, ADRP, TIP47), which regulates the dynamics of lipid storage and release from these organelles [24]. Both ABHD5 and RAB18 have been involved in the recruitment of the triglycerides from cytosolic LDs: RAB18 by competing with ADRP and inducing the LD apposition to the ER [23], and ABHD5 more directly by activating a lipase at the LD surface [30]. CES1 (also called TGH, triglyceride hydrolase) and CES3 are two ER-resident lipid hydrolases [31]. Interestingly, CES1 was found in the luminal LD proteome [32] and may participate in the second step of the VLDL synthesis [31]. The screen was controlled with inclusion of known HCV entry (CD81 [33]), replication (PI4KIIIα [34]) and assembly factors (APOE [35,36]). A functional view of our candidate selection is depicted in S1 Fig and the screening procedure is illustrated in S2 Fig

**Fig 1:**
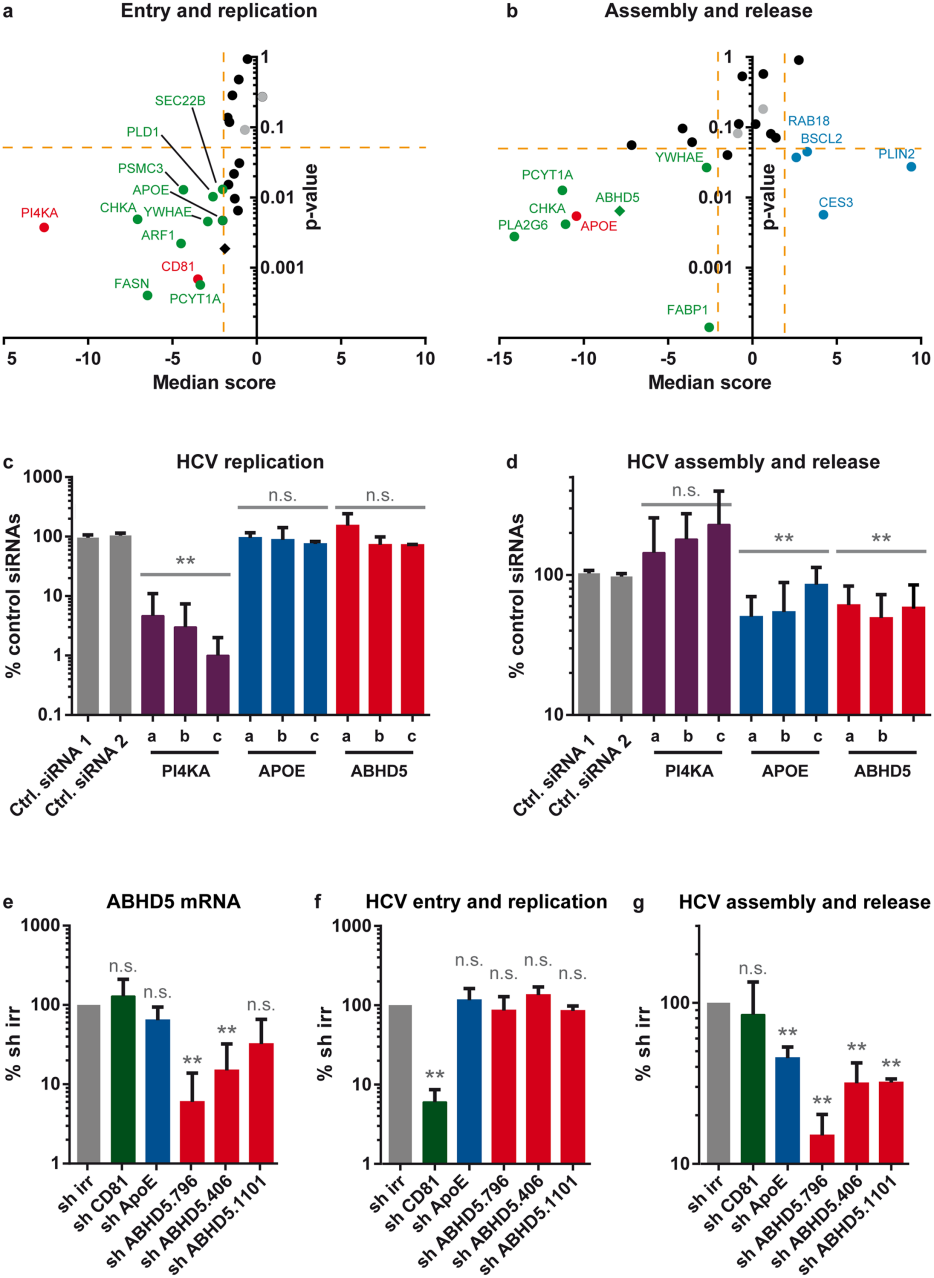
Identification of ABHD5 as a new host factor for HCV production. (**a, b**) A rational siRNA screen was designed to identify host factors involved in the lipid metabolism and participating in the HCV replication cycle (see S2 Fig) with readouts for HCV entry and replication (**a**) or assembly and release (**b**). For each graph, the p-value is plotted against the median score. A maximal p-value of 0.05 together with a mean score superior to 2 (blue dots, antiviral factors) or inferior to -2 (green dots, proviral factors) was considered highly significant. CD81, PI4KA and APOE controls are shown in red in the relevant graph and the non-targeting negative control siRNAs in grey. ABHD5 is depicted with a diamond. Yellow dotted lines indicate our statistical thresholds. (**c, d**) ABHD5-specific siRNAs used in the initial screen as a pool (panels a and b) were transfected individually into HCV RNA-transfected cells. Their specific effect on HCV RNA replication (panel **c**, corrected for cell viability effects) and progeny virion production (panel **d,** corrected for HCV RNA replication effects) is depicted after normalisation to the average value of two non-targeting siRNAs. Note that statistics were performed at the gene level. (**e- g**) Effect of ABHD5-specific shRNAs on ABHD5 gene expression (**e**), HCV entry and replication (**f**) and HCV assembly and release (**g**). (**e**) ABHD5 mRNA levels were quantified by qRT-PCR at the time of virus harvest. (**f**) HCV entry and replication were determined by the RLuc activity in the producer cell lysates at the same time point and corrected for the effects on cell viability. (**g**) The efficiency of HCV production was evaluated by the RLuc activity in target cells infected with the supernatant of shRNA-transduced and JcR-2a-infected producer cells, and corrected for the shRNA effects on HCV entry and replication.

Overall, 11 genes, including the PI4KIIIα and CD81 controls, behaved as dependency factors for HCV entry, translation or replication (Fig 1a and S1 Table). Moreover, 4 genes acted as restriction factors for HCV assembly and release, while 7 others, including the APOE positive control, were potential cofactors for HCV production (Fig 1b and S1 Table). Three primary hits were specifically contributing to HCV progeny virion production, *i*.*e*. their knockdown did not affect HCV entry and replication but significantly diminished virus assembly and release: FABP1, PLA2G6 and ABHD5. Because of its association with a human genetic disease and its interesting dual role in lipid droplet and lipoprotein metabolism, we focused here on ABHD5. Individual siRNAs and shRNAs against ABHD5 confirmed that ABHD5 expression level regulated the efficiency of HCV production while having no effect on cell viability (S2 Fig) and no or minor effects on HCV entry or RNA replication (Fig 1c–1g).

### ABHD5 mutants associated with the Chanarin-Dorfman syndrome do not support HCV production

**Fig 2:**
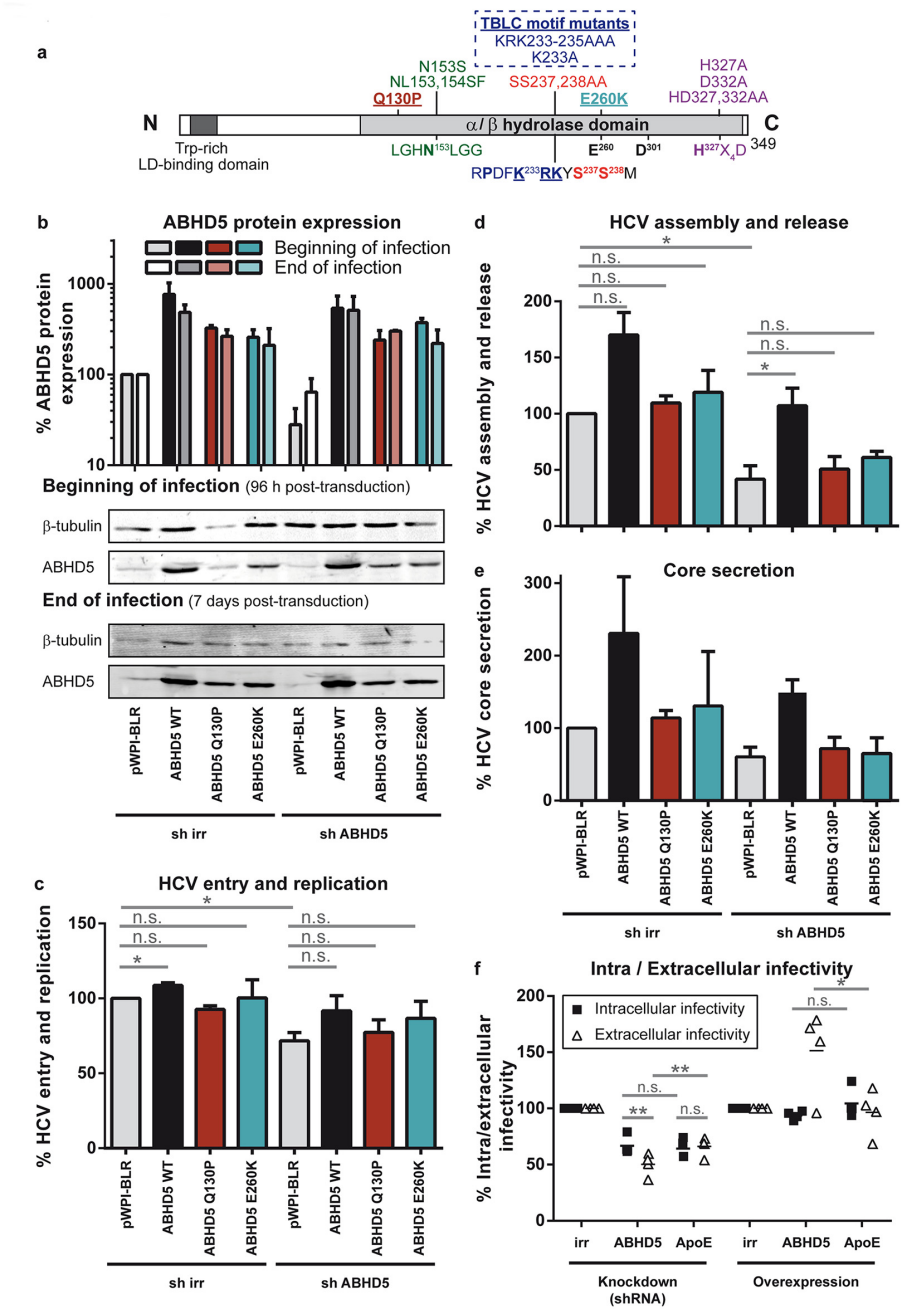
ABHD5, but not the Chanarin-Dorfman syndrome mutants, supports both HCV assembly and release. (**a**) Functional and structural organisation of ABHD5. Below are indicated the consensus sequences for the esterase/lipase motif (GXNXG, in green), and for the predicted NLS (in blue), phosphorylation sites (in red) and LPAAT motif (HX_4_D, in purple). Note that the replacement of the nucleophilic serine in the typical GXSXG lipase motif by an asparagine prevents any hydrolase activity. The predicted hydrolase pseudo-catalytic triad [16] includes residues N^153^, H^327^, and E^260^ or D^301^. The lipid droplet-binding domain is defined according to Gruber *et al*. [40]. The mutations used in this study are shown on top of the schema with a color code corresponding to their targeted motif. Those mutations associated with the Chanarin-Dorfman syndrome (Q130P, E260K) are underlined. (**b-e**) Irrelevant (sh irr) or ABHD5-specific shRNAs (sh ABHD5) were transduced into cells together with shRNA-resistant wild-type or mutant ABHD5 (Q130P or E260K), or with an empty vector (pWPI-BLR). After 4 days, the cells were infected with JcR-2a for 72 h before transferring the supernatant onto target cells. (**b**) ABHD5 expression was detected by Western blot at the beginning (96h post-transduction) and at the end of HCV infection (72 h post-infection, corresponding to 7 days post-transduction). Detection of β-tubulin served as an internal control for protein load. A representative Western blot is presented in the bottom panel while the top panel depicts the quantification of ABHD5 expression, normalised for β-tubulin expression and for the mock-treated cells (sh irr and pWPI-BLR transduction) and averaged over 3 independent experiments. (**c-e**) In the same set of experiments as in panel b, HCV replication, progeny virion production and core secretion were analysed. Note that the mild effects of ABHD5 expression on HCV entry and replication in this assay are probably due to the late time point chosen (72h post-infection) where virus spread and re-infection starts affecting the readout. (**f**) Infectious titres were determined in the supernatants (extracellular infectivity) as well as in the cleared freeze-and-thaw lysates (intracellular infectivity) of infected cells with manipulated ABHD5 or ApoE expression.

Over 30 different mutations or deletions of ABHD5 [15,16] or its promoter [37] were described in 52 patients and associated with the Chanarin-Dorfmann syndrome, a lipid storage disorder [15]. Thus, we explored whether such variants would function as HCV assembly co-factors (Fig 2). The ABHD5 Q130P and E260K CDS mutants were chosen for their broader characterisation and minimal sequence alteration [17,38]. These mutations disrupt the capacity of the protein to bind lipid droplets and ADRP [39] as well as its lipase cofactor activity [30] located in the α/β hydrolase domain of ABHD5 (Fig 2a). Western blot analyses confirmed the knockdown and the ectopic expression of wild-type or mutant ABHD5 proteins through the experiment (Fig 2b). Consistently with Fig 1, ABHD5 expression only had mild effects on HCV entry and replication (Fig 2c). Importantly, expression of the shRNA-resistant wild-type ABHD5 protein, but not of the two Chanarin-Dorfman mutants restored virus production in ABHD5-silenced cells (Fig 2d). Overexpression of the wild-type protein over the endogenous expression level promoted virus production, which was not the case for the mutants (Fig 2d). Collectively these results validate ABHD5 as novel HCV assembly co-factor and indicate that two variants of the protein involved in the Chanarin-Dorfman syndrome do not support HCV assembly.

### ABHD5 contributes to both HCV assembly and release but does not regulate the virion specific infectivity

We next aimed to specify the role of ABHD5 in HCV production. Upon manipulation of ABHD5 expression, secreted core amounts directly mirrored the released infectivity (compare Fig 2d and 2e), indicating that the virion specific infectivity was unchanged. Secondly, we compared the intra- and extracellular infectivities upon ABHD5 up- or down-regulation (Fig 2f). Knockdown of ApoE was used as a control as a specific assembly factor for HCV [13]. ABHD5 and ApoE knockdown resulted in similar reductions in intracellular infectivity, demonstrating the role of ABHD5 in virus assembly. However, contrary to ApoE, the extracellular infectivity after ABHD5 knockdown or overexpression was more affected than the intracellular infectivity, pointing at an additional role of ABHD5 in virion release.

### ABHD5 but not the CDS mutants associates with lipid droplets and the Golgi apparatus and colocalises with the HCV assembly machinery

**Fig 3:**
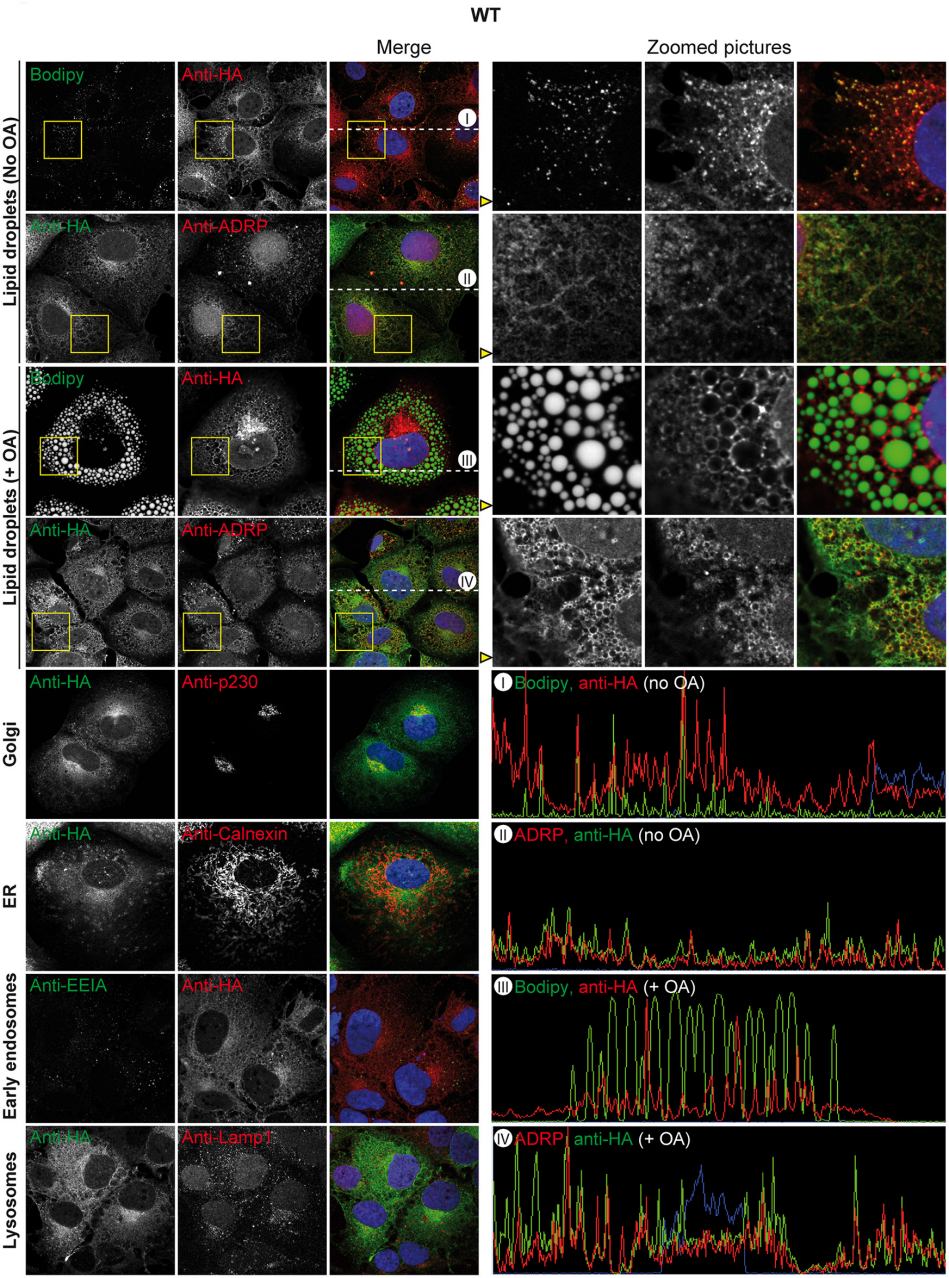
Subcellular localisation of ABHD5. Lunet N hCD81 cells expressing HA-tagged ABHD5 were stained for the HA epitope as well as for diverse organelles (LD (Bodipy or ADRP), *trans*-Golgi (p230), ER (calnexin), early endosomes (EEIA), lysosomes (Lamp1)) and counterstained with Dapi. In each panel, representative pictures are shown on the left part. For the colocalisation analysis with the lipid droplet markers Bodipy and ADRP, a portion of the image highlighted with a yellow square is magnified on the right side and depicted in the different channels in the same order. Moreover, for the same pictures, the intensity profile of the different dyes along the dotted line in the merged picture is shown in the lower right quarter and labelled with a Roman number.

**Fig 4:**
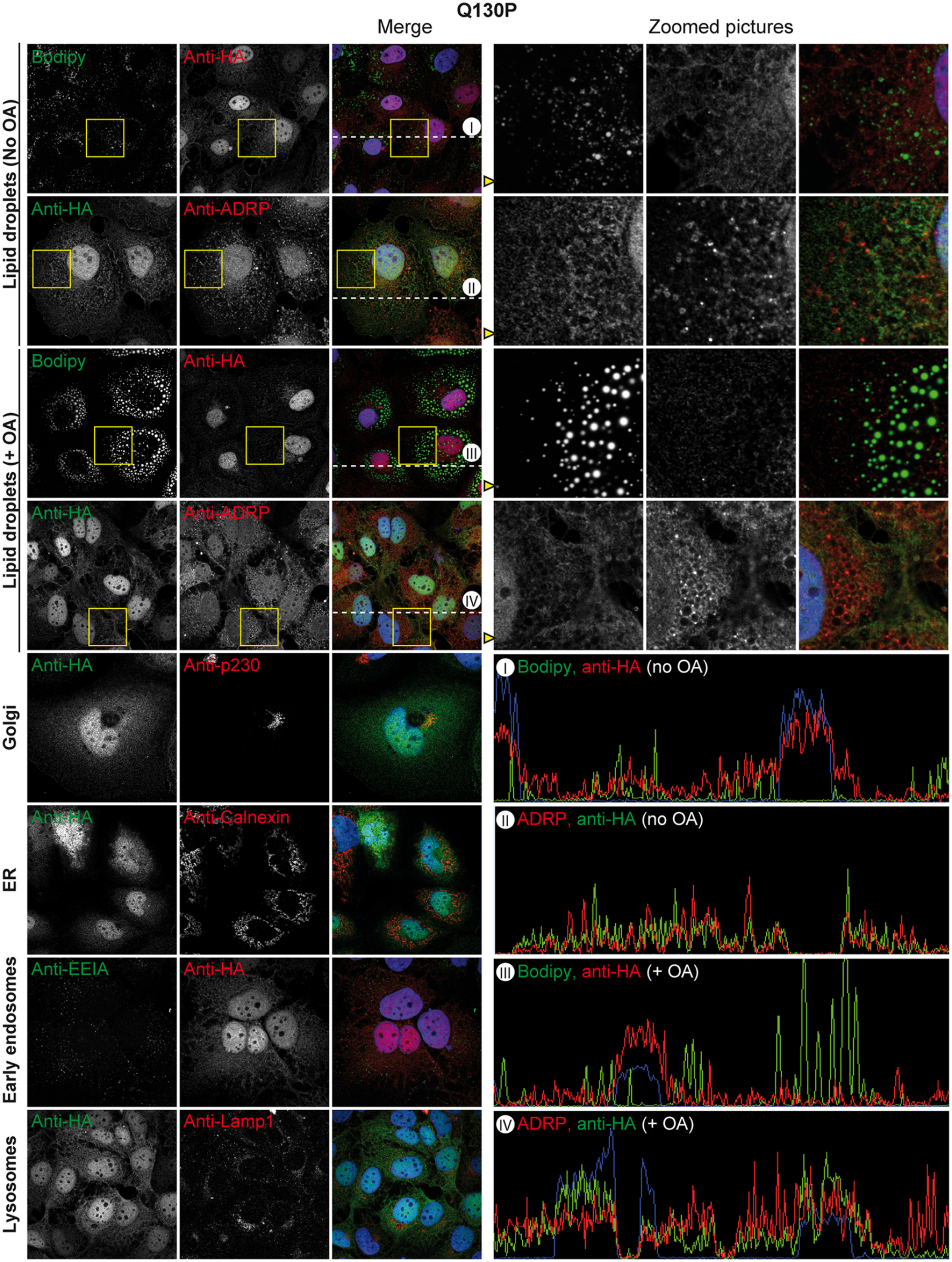
Subcellular localisation of ABHD5 Q130P Chanarin-Dorfman mutant. Lunet N hCD81 cells expressing HA-tagged Q130P mutant were stained and depicted as in Fig 3. Note that data for the E260K mutant is gathered in S4 Fig.

We next studied the localisation of ABHD5 and of the CDS mutants in our HCV permissive Huh-7-derived Lunet N hCD81 cell line by lentiviral transduction of HA-tagged ABHD5 constructs. Importantly, the HA-tagged protein shared a similar localisation pattern with the overexpressed untagged ABHD5 protein detected with an anti-ABHD5 antibody (S3 Fig). We did not observe a colocalisation with the ER marker calnexin, nor with early endosomes or lysosomes (Fig 3). However, a fraction of ABHD5 concentrated at the surface of the lipid droplets (marked with the Bodipy neutral lipid dye or the peripheral LD-associated protein ADRP), while another colocalised with the *trans*-Golgi marker p230. As already reported [18], the LD-associated ABHD5 population increased upon oleic acid treatment of the cells (Fig 3). In contrast to the wild-type protein, the CDS mutants showed a diffuse localisation through the cell, and a nuclear accumulation (Fig 4 and S4 Fig). Indeed, their LD-association was strongly reduced, even upon oleic acid induction. Nevertheless, their Golgi accumulation was at least partially preserved.

**Fig 5:**
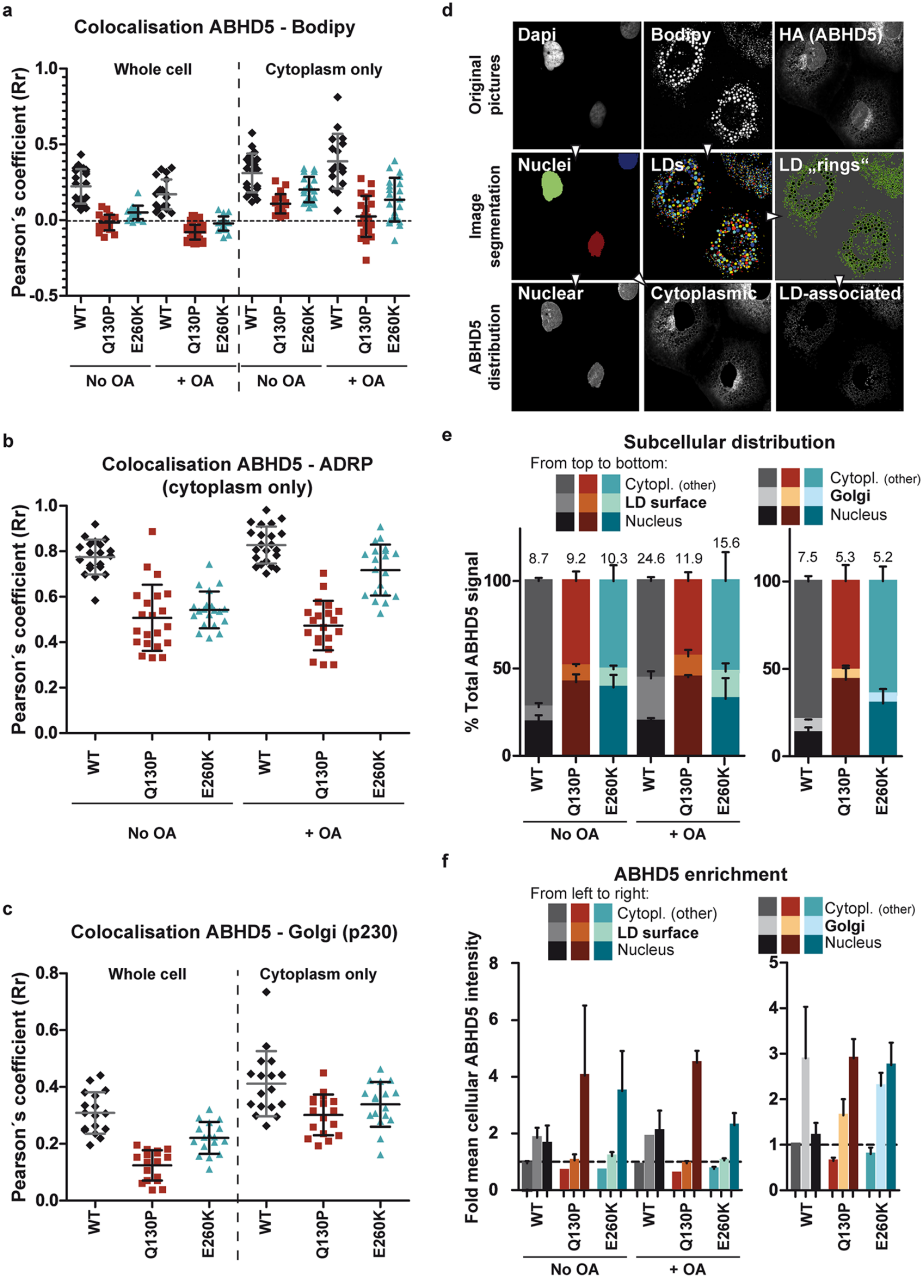
ABHD5 mutants accumulate in the nucleus and poorly associate with lipid droplets. Experimental details and representative immunofluorescence pictures are to be found in Figs 3, 4 and S4. For each analysis, the data were averaged over 2 independent experiments and at least 10 (ADRP, Bodipy) or 7 frames (Golgi) per experiment. (**a-c**) Pearson’s correlation coefficient (Rr) between HA-tagged ABHD5 and lipid droplet (**a, b**) or *trans*-Golgi markers (**c**) were calculated on the whole cells or specifically on the cytoplasm, as indicated. Note that the correlation with the ADRP marker was restricted to the cytoplasmic signal to prevent the bias that would have been caused by the diffuse, non-LD-associated, nuclear ADRP signal. Each dot corresponds to one frame. (**d-f**) Analysis of relative ABHD5 subcellular distribution and enrichment. (**d**) Cell segmentation strategy: example for the lipid droplet and nucleus segmentation. Within a multichannel picture (Dapi, Bodipy, HA-tagged ABHD5), nuclei and lipid droplets were automatically identified in the respective channels as objects. The lipid droplet periphery was extrapolated as rings of constant thickness around each individual LD. Based on this automated object identification, the ABHD5 signal was then segmented into its nuclear, cytoplasmic and LD-associated fractions. Total and mean intensities in the different cell compartments were measured and normalised to the total or mean ABHD5 intensity in the whole cell, giving the subcellular distribution and enrichment of ABHD5 across these compartments, respectively. A similar strategy was used for the Golgi segmentation (Dapi, p230, HA-tagged ABHD5 triple staining). (**e**) Subcellular distribution of ABHD5. Left panel: The total ABHD5 signal in the nucleus, at the lipid droplet periphery (LD “rings”) and in the rest of the cytoplasm was quantified and normalised to the total ABHD5 signal. The percentage of LD-associated ABHD5 is indicated on top of each bar. Right panel: A similar analysis was performed on cells stained for the Golgi apparatus instead of the lipid droplets. The percentage of Golgi-associated ABHD5 is indicated on top of each bar. (**f**) Subcellular enrichment of ABHD5. Left panel: The mean ABHD5 signal intensity in the nucleus, at the lipid droplet periphery (LD “rings”) and in the rest of the cytoplasm was quantified and normalised for the mean ABHD5 signal intensity in the whole cell (set to 1 and highlighted with a dotted horizontal line). Right panel: A similar analysis was performed on cells stained for the Golgi apparatus instead of the lipid droplets.

**Fig 6:**
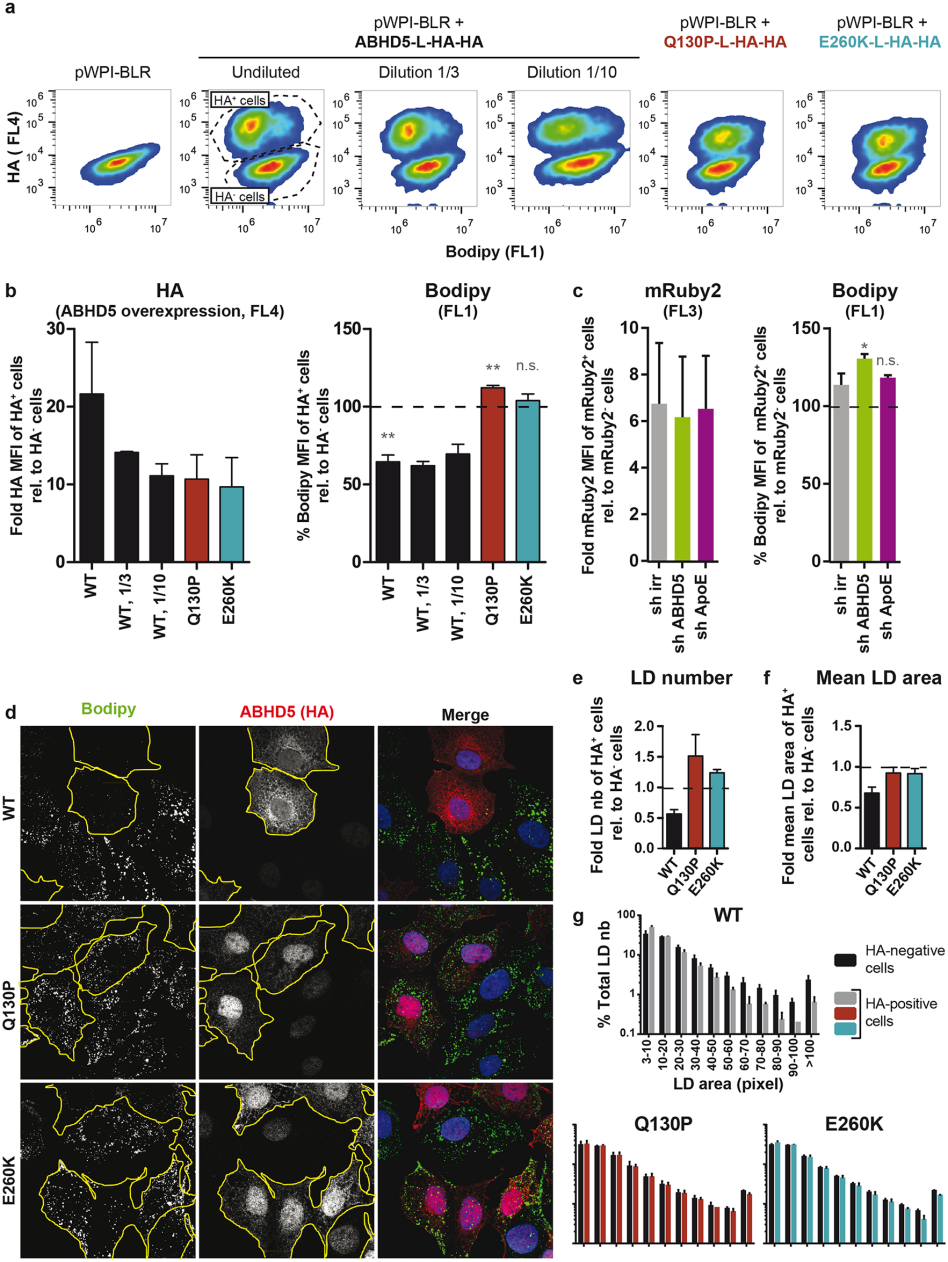
ABHD5 expression regulates the lipid droplet content of the hepatoma cells. (**a-c**) FACS readout. (**a**) Representative FACS plots for the overexpression setup. Lunet N hCD81 cells were mock-transduced (pWPI-BLR) or transduced with an HA-tagged ABHD5 construct. Mock- and ABHD5-transduced cells were mixed and stained for their lipid droplets with the Bodipy dye (FL1, x axis) and for ABHD5 overexpression with an anti-HA antibody (FL4, y axis). Fluorescence at the single cell level was analysed by FACS. The first plot shows the mock-transduced cells alone. The other six plots depict ABHD5-overexpressing cells mixed with mock-transduced cells, which serve each time as an internal reference. The ABHD5-overexpressing cells (top clouds of cells) can easily be distinguished by the HA epitope staining (FL4, y axis). Note, specifically for the wild-type-expressing cells (plots 2 to 4), the shift in the top cloud shape towards lower Bodipy intensities (FL1, x axis). To reach similar expression levels as compared to the ABHD5 mutants, wild-type ABHD5 construct was transduced with undiluted, 3 fold- or 10 fold-diluted lentiviruses. (**b**) For each ABHD5 construct, the level of ABHD5 overexpression (left panel) was estimated by the HA mean fluorescence intensity (MFI) of the HA-positive cell population (top cloud of cells) relative to the MFI of the HA-negative cell population (bottom cloud). The lipid droplet content of ABHD5-overexpressing cells (right panel) was estimated by the Bodipy MFI of the HA-positive cells after normalisation with the Bodipy MFI of the HA-negative cells. (**c**) Knockdown setup. In this setup, cells were transduced simultaneously with the indicated shRNA (sh irr, sh ABHD5, or sh ApoE) and an mRuby2-expressing construct. As in the overexpression setup, each cell population was mixed with mock-transduced cells (irrelevant shRNA + empty pWPI-Puro vector), which served as an internal reference. The expression of mRuby2 allowed distinguishing the 2 cell populations. The mixed cell populations were also stained with Bodipy to assess their lipid droplet content. The fold increase in mRuby2 expression between the shRNA- and mRuby2-transduced cells on one hand and the mock-transduced cells on the other hand is depicted in the left panel. The effect of the shRNA on the lipid droplet content (right panel) was assessed by normalising the Bodipy MFI of the mRuby2-positive cells by the MFI of the mock-transduced cells. (**b, c**) Averages and standard deviations were calculated over 3 independent experiments. (**d-g**) Microscopy readout. (**d**) Representative immunofluorescence pictures. Lunet N hCD81 cells expressing HA-tagged ABHD5 were mixed together with mock-transduced cells and reseeded on coverslips. The next day, cells were fixed and stained for the HA epitope (ABHD5 overexpression), lipid droplets (Bodipy) and nuclei (Dapi). The outline of ABHD5-overexpressing cells was drawn manually and is shown in yellow. Each picture was taken to contain both HA-positive and HA-negative cells so that the mock-transduced cells can serve as an internal control for the subsequent quantifications. (**e**) Lipid droplet number. Note that in each frame, the number of lipid droplets in HA-negative and HA-positive cell populations was normalised for the total nuclear area of the respective population, as a substitute for the whole cell area. (**f**) Mean lipid droplet area. (**e, f**) In each condition (WT / Q130P / E260K), the lipid droplet number and mean area were normalised for those calculated in the mock-transduced cells detected in the same frames. (**g**) Lipid droplet size distribution. Individual lipid droplets in HA-negative and HA-positive cells were classified in groups depending on their size. For each frame, the number of lipid droplets per size group was normalised by the total number of lipid droplets in the relevant cell population. Note that a lower diameter cut-off value of 2 pixels was used for the lipid droplet identification, so that lipid droplets with an area of less than 3.14 pixels are not detected. For space reasons, the axis labels in the 2 lower panels were not duplicated but are similar as for the WT. (**e-g**) Averages and standard deviations were calculated over 3 independent experiments and 10 frames per experiment.

The defect in LD- and *trans*-Golgi-association of the CDS mutants was further evidenced by their decreased Pearson’s correlation coefficient with both lipid droplet markers (Bodipy and ADRP, Fig 5a and 5b) and with p230 (Fig 5c), even when selectively the cytoplasmic fraction of ABHD5 was considered (Fig 5a and 5c). To complement this analysis, we quantified ABHD5 subcellular distribution using an automated imaging-based cellular segmentation method as depicted in Fig 5d. This approach quantifies the total signal intensity of ABHD5 within the given compartments and revealed a ca. two-fold nuclear accumulation of both CDS mutants compared to the wild-type as well as a slight reduction of the *trans*-Golgi-associated population (Fig 5e, right panel). ABHD5 abundance at the lipid droplet surface increased ca. 2.8-fold upon oleic acid treatment for the wild-type but only 1.3 to 1.5-fold for the CDS mutants. Consequently, the lipid droplet-associated population was reduced for the CDS mutants as compared to the wild-type in lipid-stimulated cells. Note that overexpression of wild-type ABHD5 but not the mutants reduced the size of the lipid droplet compartment (see below and Fig 6) likely introducing a calculation bias that masked this effect in the basal conditions. To overcome this sampling bias, we calculated the concentration of the ABHD5 signal intensity in the different compartments as compared to the mean ABHD5 signal intensity in the whole cell (Fig 5f). Importantly, only the wild-type protein was enriched (1.8–1.9 fold) at the lipid droplet surface—both under basal and oleic acid treatment conditions. The lipid droplet and Golgi enrichment of ABHD5 is reminiscent of the sites for HCV assembly and release [10]. Consistently, ABHD5 colocalised with the HCV assembly machinery in HCV-infected cells (S5–S7 Figs), in particular with core at the lipid droplet surface, with the host factor ApoE at the Golgi apparatus and with E2 and NS5A in the rest of the cytoplasm. This overlap was decreased or abolished for the CDS mutant Q130P. Finally, ABHD5 localisation and the aberrant distribution of the CDS mutants were confirmed in live cells (S8 Fig and S1 and S2 Videos).

### ABHD5 promotes the consumption of lipid droplets

Next, we tested the effect of ABHD5 expression on the intracellular triglyceride storage (Fig 6). Lipid droplet content was assessed by Bodipy staining and FACS (Fig 6a, 6b and 6c) or microscopy (Fig 6d–6g). To circumvent the sample-to-sample staining variability, we implemented a mixed cell population assay: ABHD5-overexpressing or depleted cells were systematically mixed with mock-transduced cells and distinguished by ABHD5 staining with the HA epitope (overexpression setup) or by co-transduction of ABHD5 shRNA with an mRuby2-encoding construct (knockdown setup). This experimental strategy is illustrated in S9 Fig

FACS analysis readily distinguished the population of ABHD5-overexpressing cells from the mock-transduced cells (HA^+^ vs. HA^-^ cells, Fig 6a) thus permitting a direct quantitative comparison of lipid droplet content (Bodipy signal) between these two cell populations. Overexpression of wild-type ABHD5 reduced the lipid droplet content of the cells by around 35%, while overexpression of the CDS mutants, even at similar expression levels, had no effect or a slight opposite effect on the lipid droplet content (Fig 6a and 6b). Thus, wild-type but not mutant ABHD5, seemed to consume lipid droplets. These results were reinforced by a significant increase in Bodipy staining upon ABHD5 knockdown (Fig 6c, right panel). Of note, possible discrepancies between mRuby2 and shRNA expression (transduced on separate vectors) might underestimate the effects observed in this last assay.

Fluorescence microscopy was used to explore ABHD5-mediated lipid droplet lipolysis at the single lipid droplet level. Each picture analysed comprised ABHD5-overexpressing cells (detected with the HA epitope tag, highlighted with a yellow outline) and mock-transduced cells (internal control) (Fig 6d). The lipid droplet content of these two cell populations was quantified in an automated fashion. In line with the FACS results, cells overexpressing a functional ABHD5 protein showed a decrease in both the number (Fig 6e) and mean area (Fig 6f) of detectable lipid droplets, with a global shift of the lipid droplet pool towards small or undetectable lipid droplets (Fig 6g). In contrast, overexpression of the Chanarin-Dorfman mutants had no effect on the cell lipid droplet content.

### Determinants responsible for ABHD5 function in LD consumption and HCV production

To determine the requirements for ABHD5 LD consumption and its relevance for HCV assembly, we generated a panel of ABHD5 mutants targeting characterised or putative ABHD5 functional domains (Fig 2a). These included ABHD5 predicted protein kinase A consensus sequence RKYSS [41] and C-terminal LPAAT motif. We further detected a potential nuclear localisation signal (NLS) corresponding to the (P/R)XXKR(K/R) motif (see Methods) and tested whether its mutation could prevent the nuclear accumulation and reverse the phenotype of the Q130P CDS mutant. Last, we reconstituted a typical lipase catalytic triad by restoring a serine residue in the lipase motif (N153S). An alternative mutant (NL153,154SF) was designed to recreate the lipase motif of a plant homolog of ABHD5, which is a weak hydrolase *in vitro* [17]. These latter mutants were constructed to assess whether a direct lipase activity of ABHD5 could be restored, bypass the requirement for the activation of a lipase to support LD hydrolysis and/or HCV production and therefore compensate for the CDS mutation. All variants were fused to a double HA tag as before.

**Fig 7:**
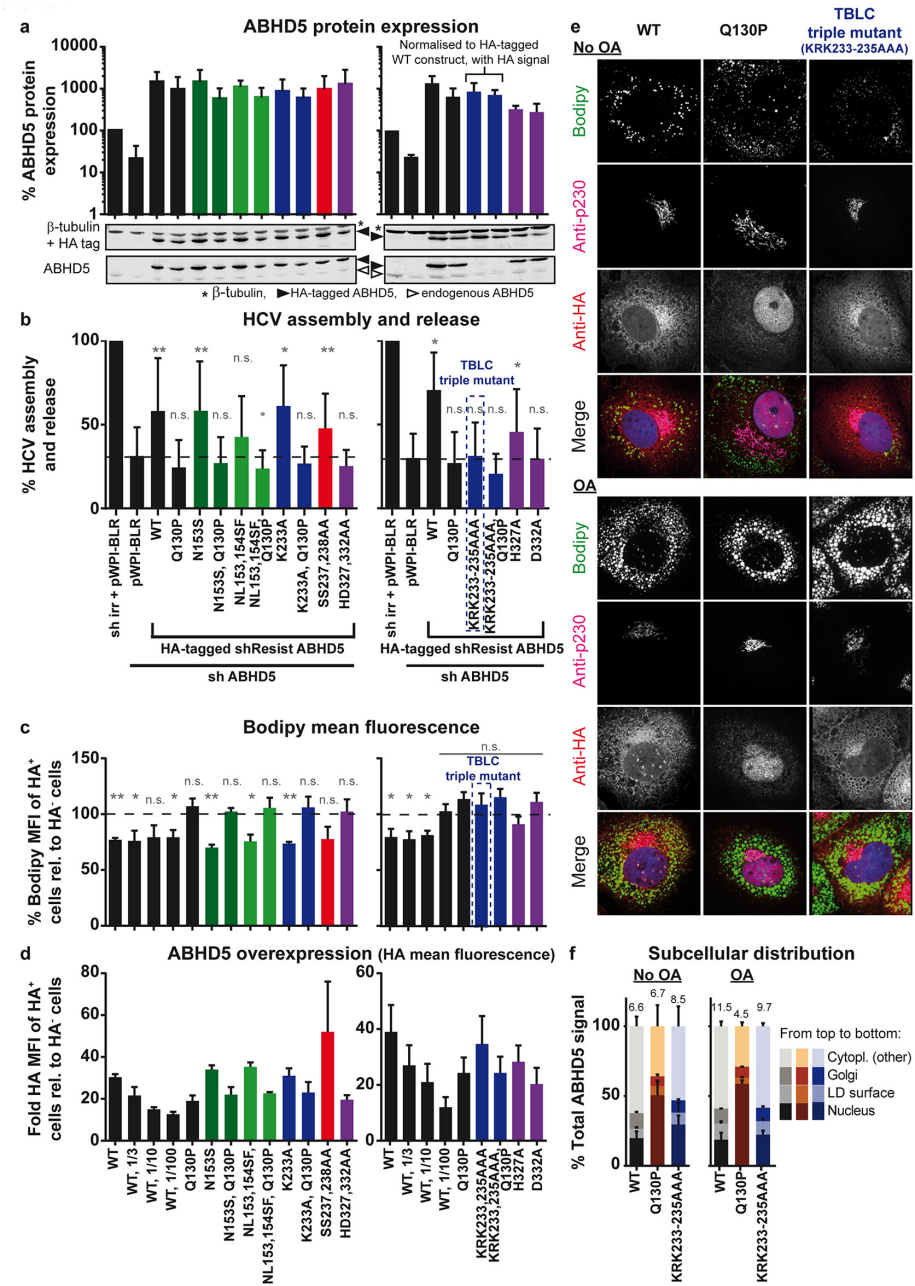
Mapping of ABHD5 determinants responsible for ABHD5 function in lipid metabolism and role in HCV virion production. ABHD5 functional domains were dissected with a panel of mutants targeting its lipase motif (green), putative NLS (blue), phosphorylation sites (red) and LPAAT motifs (purple) (see Fig 2a). All mutants were cloned with a C-terminal double HA tag. Some mutants were also combined with the Q130P CDS mutation to test for compensatory effects. (**a**) ABHD5 expression was detected by Western blot at the time of virus harvest with an anti-ABHD5 antibody and with the anti-HA tag antibody. Detection of β-tubulin served as an internal control for protein load. Note that the detection was performed with the Odyssey system, with anti-ABHD5 antibody detected in one channel and both anti-HA and anti-β-tubulin antibodies in the second channel. A representative Western blot is presented in the bottom of each panel while the top part depicts the quantification of ABHD5 expression, normalised for β-tubulin expression and for the mock-treated cells (sh irr and pWPI-BLR transduction) and averaged over the 5 (left panel) or 3 (right panel) independent rescue experiments. Note that in the right panel, the two constructs harbouring a triple mutation of the tribasic motif could not be detected with the anti-ABHD5 antibody. Their expression was therefore measured with the HA tag staining and calculated relatively to the expression of the HA-tagged wild-type construct. (**b**) Progeny virion production was analysed in the same set of experiments as in panel a (5 or 3 independent experiments for the left and right panels, respectively). Statistically significant differences were tested by comparing the overexpression of the different mutants with the knockdown control (second bar). (**c, d**) The lipolytic activity of the HA-tagged ABHD5 mutants was analysed by overexpression and with the FACS-based assay described in Fig 6a. Normalised Bodipy signals are plotted in (**c**) as percentages and the fold increases in HA epitope detection over background are depicted in (**d**). As in Fig 6, serial dilutions of the WT ABHD5 lentiviruses were included so as to reach expression levels similar to the least expressed mutants. Data show the averages over 3 independent experiments. (**e**) Lunet N hCD81 cells expressing the HA-tagged ABHD5 TBLC motif mutant or the wild-type or CDS controls were fixed and stained for the HA epitope as well as lipid droplets (Bodipy) and the Golgi (p230) and counterstained with Dapi. Representative pictures of two independent experiments are shown. (**f**) The subcellular distribution of ABHD5 was analysed as in Fig 5 (but with quadruple staining pictures) for the mutants depicted in panel e, in absence or presence of oleic acid. The numbers on top of each bar correspond to the percentage of LD-associated ABHD5. The data correspond to the average of 2 independent experiments with 10 frames per experiment and construct. (**e, f**) Note that the complete set of representative immunofluorescence pictures and quantified data covering the entire panel of mutants is available in S10–S12 Figs.

Expression of the mutants was confirmed by Western blot (Fig 7a) and their proviral function was tested in a knockdown complementation experiment (Fig 7b). Briefly, reconstitution of a typical lipase motif (N153S or NL153,154SF), single point mutation of the putative NLS (K233A) or targeting the putative phosphorylation sites (SS237,238AA) did not affect the properties of ABHD5 in this assay. More interestingly, the double mutation of the LPAAT motif abrogated ABHD5 proviral effect, while specific replacement of the histidine residue was tolerated to some extent. Triple mutation of the putative NLS did not restore the function of the CDS mutant (Q130P). In fact, this mutation abrogated the proviral effect of parental ABHD5 suggesting that this tri-basic motif is critical for ABHD5´s role in HCV assembly and possibly also its natural function. Strikingly, the capacity of the ABHD5 mutants to support HCV production correlated perfectly with their lipolytic activity (compare Fig 7b to 7c). In particular, restoration of the conserved lipase motif failed in reconstituting an active lipase. This correlation extended to the mutant subcellular localisation (S10–S12 Figs): all functional mutants localised like wild-type ABHD5, while all non-functional mutants spread as the CDS mutants and failed to associate with the lipid droplets. The single mutation of the LPAAT motif (H327A) showed an intermediate phenotype in HCV assembly, LD lipolysis and localisation pattern with partial nuclear accumulation (Fig 7 and S10–S12 Figs) thus further extending this direct correlation. The sole exception to this correlation was the KRK233-235AAA mutant of the tri-basic motif, a putative NLS, which did not support HCV assembly (Fig 7b) and LD lipolysis (Fig 7c) despite a subcellular localization comparable to parental ABHD5 (Fig 7e and 7f). Note that this mutation of the basic stretch did not prevent the nuclear localisation of the CDS mutant (Q130P) (S10–S12 Figs), arguing against its function as an NLS. This showed that lipid droplet accumulation was not sufficient for ABHD5 lipolytic or proviral activities. This is also the first description of an ABHD5 element that specifically determines the protein lipolytic activity without affecting the protein localisation. For this reason, we named this new motif the tribasic lipid droplet consumption motif (TBLC). In summary, this panel of mutants suggested that the lipid droplet degradation triggered by ABHD5 was the supporting mechanism for ABHD5 proviral function and identified the TBLC motif as a crucial determinant in this process.

## Discussion

HCV usurps the host lipid metabolism at several steps of its replication cycle. Our rationale siRNA screen highlights and extends these intricate connections: out of the 19 gene candidates, 5 were specifically involved in HCV entry or replication, 7 in assembly or release and 3 in both early and late virus replication steps (Fig 1). To our knowledge, 9 of these factors have never been associated with HCV pro- or antiviral effects in cell culture (ABHD5, CES3, FABP1, PCYT1A, PLA2G6, PLD1, PSCM3, SEC22B and YWHAE). Interestingly, the 3 hits specifically involved in HCV assembly and release (PLA2G6, ABHD5 and FABP1) gathered in the glycerolipid metabolism cluster in a bioinformatic analysis or, in the case of ABHD5 and FABP1, have been reported to interact [42] (S1 Fig). Of note, FABP1 is involved in the life cycle of *Plasmodium falciparum* [43], a parasite sharing several other host factors with HCV (*e*.*g*. CD81 and SR-BI). Moreover, PLA2G6 is a phospholipase related to PLA2G4, which we previously reported as crucial for HCV assembly [44]. CHKA and PCYT1A were additional hits belonging to the same functional cluster and both take part in the synthesis pathway for phosphatidylcholine, a crucial constituent of membranes and VLDL particles [45]. This suggests that these factors might act cooperatively in a shared cellular and proviral function and that other HCV assembly cofactors might be found by further exploring this pathway. Collectively, these observations highlight the pivotal role of lipid remodelling processes for production of infectious HCV particles.

One of the best hits of our screen was ABHD5. ABHD5 knockdown reduced HCV production to levels similar to those achieved after downregulation of ApoE. This is quite remarkable since ApoE is a constituent of the virion involved both in virus assembly and entry, while ABHD5 likely does not have a structural role in the morphogenesis but rather acts via its lipase cofactor activity. ABHD5 concentrated together with HCV core protein at the lipid droplet surface (Figs 3 and 5). It promoted the lipid droplet consumption and HCV infectious virus production at the assembly and virus release steps. Interestingly, mutations of ABHD5 associated with the Chanarin-Dorfman syndrome prevented the protein association with lipid droplets (Figs 4, 5 and S3), abrogated its lipolytic activity (Fig 6) but also its proviral function (Fig 2). Consequently, the CDS patients might exhibit some resistance towards HCV infection; the rarity of the disease [37] however precludes epidemiological studies. Over our panel of mutants targeting different ABHD5 regions, the proviral and lipolytic functions of ABHD5 always correlated (Fig 7). Although the LPAAT motif was important in ABHD5 functions (HCV assembly and LD-consumption), this is unlikely due to a genuine LPAAT activity as mutation of the putative LPAAT catalytic histidine residue only attenuated these functions (Fig 7).

In hepatocytes, ABHD5-triggered lipolysis is thought to provide lipids for VLDL synthesis [46]. VLDL morphogenesis probably occurs in two stages [47]: (i) budding of a dense lipoprotein precursor by lipidation of ApoB at the ER membrane; and (ii) modification of this precursor by fusion with a luminal lipid droplet and acquisition of exchangeable apolipoproteins such as ApoE. The proposed role of ABHD5 in mobilising the LD triglycerides for the formation of the luminal lipid droplet [18] and the fact that HCV production does not require a complete VLDL synthesis pathway [13,14] but uses ABHD5 suggest that HCV hijacks the second step of the VLDL morphogenesis rather than the precursor biogenesis for assembly and release of infectious progeny [47]. HCV might bud together with luminal lipid droplets or interact (and eventually fuse) with them in the secretory pathway. This could be essential for assembly of infectious intracellular particles. The additional effect of ABHD5 on release efficiency could have several explanations: (i) ABHD5-mediated lipid loading of the virion might be a rate-limiting step and virions that complete this process too slowly might fail secretion, (ii) poorly infectious intracellular virions that have been incompletely loaded with lipids might be retained intracellularly due to strict quality controls, (iii) the extent of ABHD5-dependent virion lipid loading might regulate the virion scission event, which could be overcome artificially by the freeze and thaw procedure used to harvest intracellular viral particles; but one also cannot exclude that (iv) ABHD5, possibly thanks to its Golgi fraction, plays an additional and independent role during the exit process.

Overall, our results indicate that the key function of ABHD5 in HCV production is its lipase cofactor activity. The identity of the activated lipase however remains elusive. While ABHD5 was shown to activate ATGL in adipocytes [30], this is still questioned in hepatocytes and a body of evidence suggests that ABHD5 cooperates with alternative or additional partners in the liver [16,17]. Importantly, we describe a new tribasic lipid droplet consumption motif (TBLC) that dissociates for the first time ABHD5 lipolytic function from its lipid droplet association [17,40]. Triple mutation of the TBLC motif (KRK233-235AAA) is likely to specifically abrogate ABHD5 interaction with his partner lipase and therefore this mutant will be a precious tool for the identification of ABHD5 effector lipase(s) in hepatocytes.

In summary, our findings indicate that ABHD5 supports HCV production by triggering the mobilisation of the lipid droplet lipid stores for the assembly and release of infectious lipo-viro-particles. They shed light on host determinants of HCV and VLDL morphogenesis, on the role of ABHD5 in hepatocytes and the etiology of the liver dysfunctions observed in the Chanarin-Dorfman patients.

## Methods

### Cell and virus culture

Lunet N hCD81 cells (generated in our lab [49]) (hCD81 matches the primary sequence of human TAPA-1, GenBank no. M33680, except for one amino acid exchange (K52M) [48]) have been described previously [49]. Lunet N hCD81 FLuc cells were derived from these Lunet N hCD81 cells by lentiviral gene transfer of a firefly luciferase (FLuc) gene using the pWPI-FLuc-BLR construct (see below). These cells as well as Huh-7.5 [50] (kindly provided by Charles Rice, Rockefeller University) and HEK 293T cells [51] (American Type Culture Collection, ATCC CRL-3612) were grown at 37°C and with 5% CO_2_ in Dulbecco’s modified Eagle’s medium (DMEM; Invitrogen) supplemented with 2 mM L-glutamine, nonessential amino acids, 100 U/ml of penicillin, 100 μg/ml of streptomycin, and 10% fetal calf serum. Blasticidine was further added at 5 μg/ml to maintain expression of the CD81 receptor and the FLuc reporter gene in the respective cell line.

For the shRNA knockdown and rescue experiments, lentiviruses were produced as previously reported [52]. Note that independent lentiviral stocks were used in the biological replicates. Stocks of JcR-2a [34] and Jc1 [53] viruses were obtained by electroporation of the *in vitro* transcribed viral RNA into Huh-7.5 cells. Electroporation and *in vitro* transcription protocols have been described elsewhere [52].

### Plasmid constructs

Three shRNAs targeting ABHD5 were constructed in the pLenti3_U6_ECEP7 expression vector, a kind gift from T. Von Hahn and S. Ciesek (Hannover Medical School). The seeding sequence of the first shRNA (shABDH5.796) was derived from the best siRNA used in the initial screen (siRNA b in Fig 2) and elongated with 2 nucleotides downstream (seeding sequence starting at position 796 in ABHD5 coding sequence: GGCCTGATTTCAAACGAAAGT). The seeding sequences for the second and third shRNAs were the two best hits predicted by the Invitrogen design tool (http://rnaidesigner.invitrogen.com/rnaiexpress/): GCACCAACAGACCTGTCTATG (position 406) and GCAGATCAACCAGAAGAATTC (position 1101). All 3 shRNAs were generated by annealing the complementary primers in a thermocycler (5 min at 90°C, ramp rate of 0.1°C /sec down to 37°C, 1h at 37°C, pause at 4°C) and ligating the resulting fragment (with overhangs) into the pLenti3_U6_ECEP7 vector between the EcoRV and PstI restriction sites. Further details on the vector used and the cloning strategy have been previously reported [54]. The control constructs expressed CD81-specific [55], ApoE-specific [44] or irrelevant shRNAs [56] in the same vector.

The Ultimate ORF clone for ABHD5 (Clone ID IOH10127, accession NM_016006.4) was obtained from Invitrogen in the pENTR(tm)221 vector. The ABHD5 sequence was subcloned into the pWPI vector with the simultaneous introduction of 4 silent mutations in the seeding sequence of shABHD5.796 (construct named “ABHD5 WT”). This shRNA-resistant ABHD5 construct was then further used as a template to generate the Q130P and E260K mutants, in the same pWPI vector. In all cases, the cloning procedure relied on two-step fusion PCR performed with overlapping and mutated primers and introduction of the insert in the pWPI vector between the BamHI and SpeI restriction sites.

The HA-tagged shRNA-resistant ABHD5 expression construct (pWPI-ABHD5-shResist-L-HA-HA-BLR) was cloned using the sequence- and ligation-independent cloning method as described by Li *et al*. [57]. Briefly, the untagged construct (pWPI-ABHD5-shResist-BLR) was digested with NdeI and SpeI and served as vector. The insert was PCR-amplified from a GBlock fragment (IDT) corresponding to the C-terminus of ABHD5, the linker sequence GGGGSG and the double HA tag (twice YPYDVPDYA), encased by the NdeI and SpeI restriction sites and 11 or 10 additional nucleotides respectively at the 5’ and 3’ ends. Both digested vector and insert were treated with the Taq DNA polymerase (NEB #M073S used at 0.5 U/ml) for 20 min at RT for its exonuclease activity to generate the compatible 5’ overhangs. Vector and insert were then mixed with an insert/vector molecular ratio of 2 and allowed to anneal at 37°C for 30 min. The mixture was finally transformed into DH5α. The HA-tagged ABHD5 mutant constructs were obtained by ligating the NotI–NdeI fragment cut from pWPI-ABHD5-Q130P-shResist-BLR or pWPI-ABHD5-E260K-shResist-BLR in the cut pWPI-ABHD5-shResist-L-HA-HA-BLR vector. Mutations described in Fig 7 were also introduced in this construct using two-step fusion PCR performed with overlapping and mutated primers and introduction of the insert in the pWPI vector between the BamHI and SpeI restriction sites.

The mTurquoise2 [58]-, mCitrine [59]- and mRuby2 [60]-fusion constructs were generated in two steps. First of all, we constructed C-terminal fusion vectors harbouring the gene for the fluorescent protein preceded by a short linker and downstream a multiple cloning site. These constructs were generated in the pWPI-Puro backbone and using BamHI and SpeI as restriction sites. Note that the coding sequences for the 3 fluorescent proteins together with the linker were ordered as GBlocks (IDT). Secondly, we transferred the ABHD5 sequence amplified by PCR from the pWPI-ABHD5-shResist-BLR or the pWPI-ABHD5-Q130P-shResist-BLR constructs into this cloning vector between the PmeI and AscI restriction sites and keeping the fluorescent protein coding sequence in frame. For the EGFP-tagged ABHD5 constructs, we PCR-amplified the NdeI-SpeI fragment covering the 5´end of ABHD5 (including the codon corresponding to Q130/P130 but downstream the shRNA-targeting site), a linker and the EGFP from the pEGFP-hCGI-58 construct (a kind gift of J.M. Brown (Wake Forest University)) [18]. This PCR was digested and incorporated in the above described pWPI-ABHD5-shResist-BLR vector. The fusion was then transferred into the pWPI-Puro vector using the AscI and SpeI restriction sites.

The pWPI-Nter-mRuby2-Puro plasmid used in Fig 6c was a N-terminal fusion vector were the mRuby2-coding sequence was cloned in the pWPI-Puro vector between the PmeI and AscI restriction sites. Finally, the pWPI-FLuc-BLR construct was generated by PCR amplifying the FLuc gene from the pFK-Luc-Jc1 plasmid [61] and cloning in the pWPI vector with the BamHI and MluI restriction sites.

All newly generated constructs were controlled by multiple restriction analyses and by sequencing. Primer sequences and detailed instructions for the cloning procedures are available upon request.

### Antibodies, dyes and siRNAs

Silencer Select siRNAs were purchased at Ambion (Life Technologies). The list and sequences of the siRNAs used in our screen are available upon request. Please note that the accession numbers of the genes targeted in our screen are given in S1 Table. SiRNAs targeting ABHD5 had the following sequences: (a) GGU UAA UCA UCU CAU UUU Att; (b) GGC CUG AUU UCA AAC GAA Att and (c) CGA CCA CAU UCA UAU GUG Att. Non-targeting control siRNAs 1 and 2 were also obtained from Ambion (#4390843, siRNA ID #s813 and #s814) and corresponded to the sequences UAA CGA CGC GAC GUA Att (ctrl. siRNA 1) and UCG UAA GUA AGC GCA ACC Ctt (ctrl. siRNA 2).

Mouse and rabbit anti-HA antibodies were purchased from Covance (#MMS-101P) and Sigma (#H6908), respectively. The anti-ABHD5 antibody was obtained from Tebu-bio (Abnova ABHD5 mouse monoclonal antibody, clone 1F3, #H00051099-M01). The following antibodies were used to stain cellular organelles and proteins: rabbit anti-β-tubulin monoclonal antibody (Thermo Scientific, clone E.884.5, #M45-15002), mouse anti-calnexin (ER marker, Abcam, ab31290), mouse anti-p230 (Golgi marker, BD #611281), rabbit anti-ApoE (Santa Cruz H223), FITC-conjugated mouse anti-EEIA-FITC (BD #612006), mouse anti-Lamp1 (Abcam #ab25630). The sheep anti-ADRP antibody was a kind gift from J. McLauchlan [62]. Antibodies against HCV proteins consisted of the anti-core C7-50 antibody [63], the anti-E2 CBH23 antibody [64] and the anti-NS5A 9E10 antibody [65], which were kindly provided by D. Moradpour, S.K. Foung and C.M. Rice, respectively. Bodipy 495/503 was purchased from Invitrogen.

The Alexa- and IRDye-conjugated secondary antibodies used for immunofluorescence and western blotting were from Life Technologies and LI-COR Biosciences, respectively.

### SiRNA-based functional screen

The screening procedure was derived from strategies previously published by us and others [34,66] (S2 Fig). Briefly, Lunet N hCD81 FLuc cells were seeded at 10^4^ cells/well in 96-well dishes, without antibiotics (producer cells). Transfection of the siRNA pools was performed 5 h later and overnight with the Lipofectamine RNAiMAX transfection reagent (Invitrogen) and according to the manufacturer’s instructions. Per well, 0.5 pmol of each of the three gene-targerting siRNAs (or 1.5 pmol of the negative siRNA control 1 or 2) and 0.2 μl of lipofectamine were used. The cell medium was replaced the next day. Forty-eight hours post-transfection, cells were infected overnight with the Jc-R2a virus and the medium changed. Two days later, the producer cells were lysed for Firefly luciferase (Fluc, for cell viability) and *Renilla* luciferase (RLuc, for HCV entry and replication) activity measurements. The producer cell supernatants were used to infect target Lunet-hCD81-FLuc cells seeded the day before at 3 x 10^3^ cells/well in 96-well dishes. These target cells were lysed 72 h post-infection for RLuc activity readout (whole replication cycle). The luciferase readouts were performed as previously described [67,68].

### siRNA validation assay

Lunet N hCD81 FLuc cells (producer cells) were electroporated with JcR-2a RNA as previously described [52] and seeded at 8 x 10^4^ cells/well in 24-well dishes, without antibiotics. Transfection of single siRNAs was performed 5 h later and overnight as described above, but with 2.5 pmol siRNA and 0.8 μl lipofectamine RNAiMAX per well. Ninety-six hours post-transfection, the producer cells were lysed for FLuc and RLuc activity measurements. The producer cell supernatants were cleared by centrifugation (5 min, 13,000 g, RT) and used to infect target Lunet-hCD81-FLuc cells seeded the day before at 2 x 10^4^ cells/well in 24-well dishes. These target cells were lysed 72 h post-infection for RLuc activity readout.

### Transient ABHD5 shRNA-mediated knockdown

Lunet N hCD81 FLuc cells (producer cells) were seeded at 2 x 10^4^ cells/well in 24-well dishes and transduced one day later for 4 h with shRNA-expressing lentiviruses, in duplicates. Three days post-transduction, twice one third of the cells were transferred into 12-well dishes. One day later (96h post-transduction), these cells (4 replicates for each condition) were infected overnight with a JcR-2a virus stock and the medium was replaced. Three days post-infection, the producer cells were lysed for FLuc and RLuc measurements (duplicates) or for RNA extraction (duplicates) and determination of the ABHD5 mRNA level at the end of the infection period. The cell supernatants (4 replicates) were used to infect target Lunet-hCD81-FLuc cells seeded the day before at 3 x 10^4^ cells/well in 12-well dishes. These target cells were lysed 72 h post-infection for RLuc activity readout.

### ABHD5 rescue experiments

The protocol for the rescue experiments was adapted from the transient shRNA knockdown experiment with the following modifications. Note that the time schedule for the rescue experiment was slightly shortened in Fig 7 and S8 Fig (see indications in brackets in this paragraph) as compared to Fig 2, in an attempt to optimise the knockdown efficiency and to prevent virus spread from affecting the RNA replication readout (see Fig 2c). Transduction was performed simultaneously with lentiviruses expressing the shRNA- and rescue constructs in triplicate wells. Three days post-transduction [2 days], the cells were split as follows in 12-well dishes: (i) respectively 1/3 and 1/6 of the cells were seeded for the determination of ABHD5 expression by western blot at 96h post-transduction [72h] (beginning of the infection period) and 72 h post-infection [48h] (or 7 days [5 days] post-transduction, end of the infection period); (ii) 1/6 of the cells were seeded for JcR-2a infection and determination of the efficiency of the whole HCV replication cycle as above. An aliquot of the producer cell supernatant was kept for quantification of the released core amount by ELISA with a diagnostic kit (Architect Anti-HCV; Abbott).

For Western blot analysis of ABHD5 expression levels, trypsinised cell pellets were lysed in reducing Laemmli buffer, treated with benzonase for 15 min at 37°C and heated for 5 min at 95°C before loading on a 10% gel and separation by SDS-PAGE. Proteins were transferred with a semi-dry blotter on a PVDF membrane (Millipore) and probed with mouse anti-ABHD5 (1/1,000) antibodies and the rabbit anti-HA (1/1,000, used in Fig 7) and anti-β-tubulin (1/1,000) antibodies followed by the secondary IRDye 800CW donkey anti-mouse IgG and IRDye 680RD donkey anti-rabbit IgG antibodies (both at 1/15,000, LI-COR). Signal intensities were read and quantified with the Odyssey CLx imager (LI-COR).

### Extra/ intracellular infectivity assay

Lunet N hCD81 FLuc cells were electroporated with JcR-2a and once the cells have attached (around 8 h post-electroporation) ABHD5 expression was regulated by overnight lentiviral transduction of specific shRNA or expression construct. The regulation of APOE expression was used as a control. 72 h post-electroporation, intracellular virions were released from the cells by freeze-thaw as previously described [34,66] and their infectivity assessed and compared to the released extracellular infectivity by measuring the RLuc activities in infected target cells.

### Relative quantification of ABHD5 mRNA expression by qRT-PCR

For each sample, total RNA was extracted from the producer cells at 72 h post-infection with the total Nucleospin RNA II kit (# 740955, Macherey-Nagel, Düren, Germany). ABHD5-specific qRT-PCR was performed in duplex with the actin or GAPDH calibrator in the Lightcycler 480 instrument (Roche, Mannheim, Germany), according to previously published instructions [52]. The sets of primers and probe were ordered from TIB MolBiol (Berlin, Germany) with the following sequences: F-ABHD5, 5’-AGT TTG TGG AAT CCA TTG AAG AGT G-3’; R-ABHD5, 5’-CTG CAA TCC TTA GGC CAG CTA-3’; ABHD5-TM probe, 5’-FAM-CGA GTA AGC CAA GAA TCC AC-BBQ-3’; F-actin, 5’-AGC CTC GCC TTT GCC GA-2’; R-actin, 5’-CTGGTGCCTGGGGCG-3’; actin-TM probe, 5’-YAK-CCG CCG CCC GTC CAC ACC CGC C-BBQ-3’; F-GAPDH, 5’-GAA GGT GAA GGT CGG AGT C-3’; R-GAPDH, 5’-GAA GAT GGT GAT GGG ATT TC-3’; GAPDH-TM probe: 5’-LCRed640-CAA GCT TCC CGT TCT CAG CCT-BBQ-3’. Note that for the FAM/YAK probe combination, channel overlap was corrected by color compensation according to the instructions from the Roche Lightcycler manual.

### Immunofluorescence

Lunet N hCD81 FLuc cells were seeded at 2 x 10^4^ cells/well on coverslips in 24-well dishes and transduced overnight to express HA-tagged wild-type or mutant ABHD5. For the oleic acid treatment, 30 μl BSA (Gibco, #30036–578, dissolved at 10 mg/ml) were vortexed with 1.14 μl of oleic acid (Sigma # O1008) and diluted in 10 ml medium. The mixture was added onto the cells at 48 h post-transduction and cells were fixed after an overnight incubation. For the colocalisation study between ABHD5 and HCV proteins, cells were seeded as above but first infected 24h post-seeding with Jc1 virus for 4 hours and then transduced overnight to express HA-tagged wild-type or mutant ABHD5. Cells were fixed 72h post-Jc1 infection and after an overnight oleic acid treatment if applicable.

Cell fixation and permeabilisation, antibody dilutions, immunofluorescence staining and confocal microscope observation were performed as previously described [69] but with the following modifications: antibodies were incubated for 1 h at RT, rabbit anti-HA antibody was used diluted 1/1,000, mouse anti-ABHD5 1/1,000, mouse anti-calnexin 1/2,000, mouse anti-p230 1/100, FITC-conjugated mouse anti-EEIA 1/500, mouse anti-Lamp1 1/500 and rabbit anti-ApoE 1/200. Note that coverslips were mounted with Fluoromount-G (product 100–01; Southern Biotech, Birmingham, AL) or ProLong Gold Antifade (Invitrogen), but the latter was avoided for samples stained with Bodipy 495/503, due to poor performance of the lipid dye in this mounting reagent.

Pictures were taken either on an Olympus laser-scanning confocal microscope as described before [69] or on a Nikon Ti-E microscope equipped with a Perfect Focus System (Nikon), a Yokogawa CSU-X1 spinning-disc and a cage incubator (37°C, 5% CO_2_; Okolab). A 100x magnification lens was used for all pictures except in Fig 6 where a 60x magnification lens was chosen to capture a broader field.

### Immunofluorescence quantifications

IF quantifications were performed with Fiji and CellProfiler. Pipelines elaborated with CellProfiler were systematically tested and iteratively optimised by running them on a random subset of pictures representing each tested condition. Further details on the pipelines developed and used in this study are available upon request.

### Pearson’s correlation coefficient

The Pearson’s correlation coefficient was calculated with the Mander’s coefficient or the Coloc2 plugin in Fiji. For those analyses restricted to the cytoplasmic proteins (Fig 5), nuclear signals were excluded by using a mask created manually by thresholding using the Dapi channel. To quantify the colocalisation between ABHD5 and ApoE, regions of interests (ROIs) were manually drawn in each frame to select the HA-positive cells. For the colocalisation between ABHD5 and HCV proteins, double positive cells (cells expressing both the HA-tagged ABHD5 construct and HCV proteins) were selected in a similar way.

### Subcellular distribution and enrichment of ABHD5

The multichannel pictures corresponding to the Dapi / ABHD5 (HA) / Lipid droplet and/or Golgi costaining were loaded in CellProfiler. The ABHD5 picture was thresholded to remove very low intensity pixels. Nuclei, lipid droplets and Golgi stacks were identified automatically as objects using a constant manual intensity threshold and according to their size and shape. Since ABHD5 associates to the LD periphery, this area was also identified as rings by sequentially expanding and shrinking the size of the recognised lipid droplet objects. These objects (nuclei, LD rings, Golgi) were used to mask the thresholded ABHD5 pictures and segment the ABHD5 signal into its nuclear, cytoplasmic, LD- and Golgi-associated components. Mean and total ABHD5 signal intensities in the different cell compartments and in the whole thresholded picture were measured.

### Quantification of the size and number of individual LDs

For each picture, a mask encompassing the HA-positive cells was created manually in Fiji. These masks were loaded together with the original immunofluorescence pictures in CellProfiler. Using this program, nuclei and lipid droplets were automatically identified as objects using a constant manual intensity threshold and according to their size (nuclei between 30–300 pixels diameter, lipid droplets between 2–40 pixels diameter) and shape. Taking into account the previously created masks, it was possible to distinguish lipid droplets and nuclei belonging to HA-positive or HA-negative cells. The outputs included, for each frame, the total number of lipid droplets and their mean area, the size of each lipid droplet and the total nuclear area, for each cell population. Note that in Fig 5e, the number of lipid droplets in HA-negative and positive cell populations was normalised, in each frame, for the total nuclear area of the relevant cell population. This is to account for possible differences in the ratio of HA-negative and positive cells (the nuclear rather than whole cell area was used because of the clear Dapi outlines allowing a robust and automated area calculation). In Fig 6g, for each frame and cell population, the number of lipid droplets in each size category was calculated as a percentage of the total number of lipid droplets. Data were then averaged over 10 frames (each frame containing both HA-positive and HA-negative cells) and finally over 3 independent experiments.

### ABHD5 localisation in live cells

Lunet N hCD81 cells were seeded at 2x10^5^ cells per dish in 35mm-diameter IBIDI μ-dishes and lentivirally transduced overnight for the simultaneous expression of the mTurquoise2-tagged wild-type ABHD5 and the mCitrine-tagged Q130P mutant. When relevant, oleic acid combined with BSA (see above) was added for 4 hours prior observation. Before imaging, cells were washed and the medium replaced by complete DMEM without phenol red and with 10 mM Hepes. Cells were imaged with the Nikon microscope described above, using the Apo TIRF 100x objective and at 37°C with 5% CO_2_. Acquisition was sequential with the 445nm laser and a CFPHQ emission filter on one hand (mTurquoise2 detection) and the 515 nm laser and YFP emission filter on the other hand (mCitrine detection). Z-stacks were taken with the optimal step size according to the Nyquist criteria (in this case, 200 nm) and 3D reconstitutions performed with the Nikon NIS Elements software.

### Mixed cell population assays for the quantification of the lipid droplet content

#### Assay for a parallel FACS and microscopy readout (Fig 6 and S9 Fig)

Lunet N hCD81 cells were seeded at 5x10^5^ cells per dish in 6-cm-diameter dishes and lentivirally transduced overnight for the expression of the different HA-tagged ABHD5 variants (undiluted or diluted as mentionned in the figures). Lentiviruses generated with the pWPI-BLR empty vector served to transduce the mock control cells. Two days post-transduction, the cells were harvested by trypsinisation and reseeded as mixed cell populations in 6-well dishes for the FACS readout or 24-well-dishes on coverslips (for the microscopy readout), in a way to reach confluence the next day. In other words, mock-transduced cells were mixed independently and at roughly equal cell numbers with each of the different HA-tagged-ABHD5-transduced cells. A portion of the cells were reseeded unmixed as controls. The next day, cells for FACS were harvested by trypsinisation, fixed in FACS fixation buffer (PBS-1% FCS-0,5% PFA) and stored at 4°C until staining. Cells for microscopy were treated as mentioned above.

Note that samples for the knockdown setup (Fig 6c) were treated following the same schedule as above but only analysed by FACS. In this case, cells were transduced at the same time with the indicated shRNA (sh irr, sh ABHD5, or sh ApoE) and the pWPI-Nter-mRuby2-Puro construct. Efficient mRuby2 expression was observed with at least 65-85-87% of the treated cells being strongly positive in the 3 independent experiments. Of note, it is possible that not all mRuby2-positive cells were efficiently targeted by the ABHD5 shRNA and vice versa, which would underestimate the effects observed in this assay. These cells were then mixed with mock-transduced cells (irrelevant shRNA + empty pWPI-Puro vector), which serve as an internal reference, and mixed cell populations were stained with Bodipy.

#### Adapted assay for FACS readout only (Fig 7)

In this case, the assay was slightly simplified as follows. Lunet N hCD81 cells were seeded at 2x10^5^ cells per dish in 6-cm-diameter dishes and lentivirally transduced as above. However, cells were mixed only upon harvesting, 3 days post-transduction. At this time point, cells were harvested by trypsinisation and mock-transduced cells were added to each of the HA-tagged-ABHD5-transduced cells at roughly equal cell numbers. Again, unmixed cell populations were kept as controls. The different cell samples were washed once in PBS and fixed directly in FACS fixation buffer.

### FACS staining and measurement

Fixed cells were washed twice in FACS Wash buffer (PBS-1% FCS) and permeabilised in FACS Permeabilisation Buffer (PBS-1% FCS-0,1% Saponin) for 20 min on ice. The primary antibody (mouse anti-HA) was incubated in FACS Permeabilisation Buffer for 20 min on ice. Bodipy (diluted 1/3000) and the secondary antibody (AlexaA647-conjugated anti-mouse antibody, 1/1000) were added together and in the same buffer also for 20 min on ice. Between and after the antibody incubations the cells were washed twice in FACS Wash Buffer. Finally, cells were resuspended in FACS Fixation buffer and analysed with the BD Accuri C6 flow cytometer and corresponding software. Gating was used to eliminate cell debris from the analysis. The Bodipy/A647-stained samples were analysed with the FL-1 and FL-4 channels, which did not require color compensation. For the Bodipy/mRuby2 dye combination (Fig 6c), the cell permeabilisation step was omitted and cells were stained with Bodipy only and in FACS Wash buffer. The fluorescence was read in the FL-1 and FL-3 channels and color compensation was applied according to the manufacturer´s instructions. Mean fluorescence intensities in the respective channels for the distinct cell populations were calculated by the BD Accuri C6 software. Note that the representative pseudocolor plots depicted in Fig 6a were however obtained by importing the raw Accuri C6 data into FlowJo and repeating the manual gating to eliminate cell debris.

### Nuclear localisation sequence prediction

The presence of potential NLS was analyzed with cNLS Mapper (http://nls-mapper.iab.keio.ac.jp/cgi-bin/NLS_Mapper_form.cgi). This program predicted a non-canonical importin-α-dependent monopartite NLS with the typical (P/R)XXKR(K/R) motif starting at position 229 and a score of 7 (on a scale of 1 to 10, knowing that NLS with scores of at least 8 induce the complete nuclear localisation of a GUS-GFP reporter protein) [70,71].

### Combined DAVID/STRING functional protein view

Functional annotation clustering and enrichment scoring of the 20 candidates was performed using DAVID (http://david.abcc.ncifcrf.gov) version 6.7, with ‘‘high” classification stringency settings [72]. DAVID analysis yielded 3 highly enriched clusters (EASE score above 1) and 6 clusters in total. We next integrated STRING 9.1 database interactions (http://www.string-db.org) into the DAVID functional annotation clustering using the Matlab script previously described [73]. Unclustered proteins were placed in the network as inverted arrowheads. We added STRING interactions (solid lines) with a combined confidence score of 0.8 or higher between proteins of different clusters, and interactions of 0.4 or higher between proteins within the same functional annotation cluster. STRING interactions, which were exclusively based on textmining, were excluded from the network. Proteins were placed in their approximate cellular locations manually. For selected candidates (TIP47, Seipin, ADRP) we used the annotation commonly used in the HCV field instead of the Uniprot ID.

### Statistics

Statistical data analysis was performed in R (http://www.r-project.org). siRNA screening data was analysed using the Bioconductor package RNAither [74], using lowess and negcontrol normalisation, and using p<0.05 as threshold for hit selection. Individual follow-up experiments (Figs 1c–1f and 2) were statistically analysed using Welch’s t-test or a paired t-test where applicable. For the rescue experiments in Fig 7, the raw data were first pre-processed and paired t-tests were then performed. Pre-processing of the data consisted in (i) log-transformation of the raw luciferase measurements, (ii) calculation of the ratios between these log-transformed RLU in target vs. producer cells, and (iii) correction of each individual experiment by subtracting the mean ratio of the control from the other conditions. For the FACS-based experiments, the FL1 ratios of the two cell populations were compared to the 100% control with a t-test. In all experiments, p-values of <0.05 were considered statistically significant (*), and p-values of <0.01 were considered highly significant (**).

## Supporting Information

S1 Fig Functional map of host factors tested in our siRNA screen. Functional annotation clusters (dotted boxes) and interactions (bold lines) are depicted and proteins are placed in their predominant cellular location. Proteins with proviral and antiviral function on HCV assembly and release are highlighted in green and blue, respectively. Note that a new interaction between ABHD5 and FABP1 was reported [42] but not inventoried in the STRING database yet. For this reason, this interaction was added as a grey line and was not taken into account in the functional clustering.
(TIF)

S2 Fig Strategy of the siRNA screen and effect of ABHD5 knockdown on cell viability. (**a**) The screen was conducted in Lunet N hCD81-Fluc cells, whose constitutive Firefly luciferase expression was used as a marker for cell viability. The cells were transfected with a pool of 3 siRNAs against each target gene and infected with a Renilla luciferase-reporter virus (JcR-2a). The RLuc activity in the “producer” cell lysates, once corrected for the cell viability is an indicator of HCV entry and RNA replication (see results in Fig 1a). Finally, naive Lunet N hCD81-Fluc cells were infected with the supernatant of the siRNA-transfected and HCV-infected producer cells. The RLuc activity in these “target” cells therefore reflects, once corrected for HCV entry and RNA replication, the efficiency of HCV production (see results in Fig 1b). (**b, c**) Effect of the ABHD5-specific siRNAs (panel b, data relating to Fig 1c and 1d) or shRNAs (panel c, data relating to Fig 1e, 1f and 1g) on the cell viability. Cell viability was determined by the FLuc activity in the producer cell lysates at the time of virus harvest.
(TIF)

S3 Fig Subcellular localisation of untagged ABHD5. Untagged ABHD5 was expressed by lentiviral transduction in the Lunet N hCD81 cell line. The cells were fixed 48 h post-transduction, after, when applicable, overnight induction with oleic acid (bottom row). Samples were stained with anti-ABHD5 antibody and with Bodipy.
(TIF)

S4 Fig Subcellular localisation of the ABHD5 E260K CDS mutant. The localisation of the E260K mutant was analysed the same way as for the wild-type and Q130P variant in Figs 3 and 4, respectively. This figure displays representative pictures while the phenotypes quantified over 2 independent experiments are depicted in Fig 5.
(TIF)

S5 Fig ABHD5 colocalises with HCV proteins and with ApoE. Lunet N hCD81 cells were infected with Jc1 virus and transduced to express HA-tagged wild-type ABHD5. Cells were stained for the HA epitope as well as for diverse HCV proteins or ApoE. For each picture, a portion of the image highlighted with a yellow square is magnified on the right side and depicted in the different channels in the same order.
(TIF)

S6 Fig Decrease in association of the Q130P CDS mutant with HCV proteins and ApoE. Lunet N hCD81 cells were infected with Jc1 virus and transduced to express HA-tagged Q130P mutant. Cells were stained and images presented as in S4 Fig.
(TIF)

S7 Fig ABHD5, but not the Q130P CDS mutant, colocalises with the HCV assembly machinery. (**a**) Intensity profiles of wild-type HA-tagged ABHD5 (green), Dapi (blue) and core, E2 or ApoE signals (red) across a section of the images depicted in panel a (see white dotted line). The black rectangles at the bottom of the profiles indicate the approximate position of the Golgi apparatus, as suggested by the concentration of the ABHD5 staining. (**b**) Colocalisation between HA-tagged ABHD5 and HCV proteins or ApoE was assessed with the Pearson’s correlation coefficient (Rr) calculated over 2 (E2, NS5A) to 3 (core, ApoE) independent experiments and 9–15 frames per experiment. Note that for each frame, Rr was calculated over a ROI corresponding to the double-positive cells (transduced and infected cells). Each dot corresponds to one frame.
(TIF)

S8 Fig Aberrant subcellular localisation of the Chanarin-Dorfman mutant in live cells. (**a, b**) Rescue experiment. (**a**) Western Blot analysis of the expression of the fluorescently tagged ABHD5 constructs 5 days post-transduction (end of HCV infection). Detection of β-tubulin served as an internal control for protein load. (**b**) Fluorescently tagged ABHD5 constructs support HCV assembly and release. Progeny virion production was analysed by normalising the released infectious titre by the replication values. (**c, d**) Lunet N hCD81 cells were transduced simultaneously for expression of wild-type and mutant ABHD5. Note that the wild-type construct was fused to mTurquoise2, while the Q130P mutant was fused to mCitrine. Localisation of the two fusion proteins was investigated in untreated (**c**) or oleic-acid-treated cells (**d**). Note that WT-mTurquoise2 and Q130P-mCitrine are shown in green and red, respectively. For 3 dimensional reconstitutions, please see S1 and S2 Videos.
(TIF)

S9 Fig Experimental design to study the effect of ABHD5 expression on the lipid droplet content. This figure summarises the approach used in Fig 6, with the overexpression (**a**) and knockdown (**b**) setups. Please see the legend of Fig 6 for a description of the experiment.
(TIF)

S10 Fig Subcellular localisation of the ABHD5 mutant panel. Note that together with S11 Fig, this figure reproduces and extends the data presented in Fig 7e to the complete list of mutants introduced in Fig 7. In this figure, the mutants corresponding to Fig 7a–7d, left panels, are shown. Representative pictures illustrating the subcellular localisation of the mutants, see the legend of Fig 7e for a detailed description.
(TIF)

S11 Fig Subcellular localisation of the ABHD5 mutant panel (following of S10 Fig). Note that together with S10 Fig, this figure reproduces and extends the data presented in Fig 7e to the complete list of mutants introduced in Fig 7. In this figure, the mutants corresponding to Fig 7a–7d, right panels, are shown. Representative pictures illustrating the subcellular localisation of the mutants, see the legend of Fig 7e for a detailed description.
(TIF)

S12 Fig Quantification of the subcellular localisation of the ABHD5 mutant panel. The subcellular distribution (**a, b**) and enrichment (**c, d**) of ABHD5 was analysed as in Fig 5 for some of the mutants depicted in Fig 7, in absence (**a, c**) or presence (**b, d**) of oleic acid. The numbers on top of each bar correspond to the percentage of LD-associated ABHD5. The data correspond to the average of 2 independent experiments with 10 frames per experiment and construct and representative pictures are to be found in S11 Fig.
(TIF)

S1 Video Aberrant subcellular localisation of the Chanarin-Dorfman mutant in live cells. The cell samples have been described in S8 Fig and correspond to untreated cells (-OA). The cells were imaged alive in Z-stacks for 3D reconstitution. The video shows the independent channels imaged sequentially (left, WT-mTurquoise2; middle, Q130P-mCitrine) and the merge (right).
(AVI)

S2 Video Aberrant subcellular localisation of the Chanarin-Dorfman mutant in live cells (oleic acid treated cells). The cell samples have been described in S8 Fig and correspond to oleic-acid treated cells (+OA). Imaging and video montage were performed as in S1 Video (see legend).
(AVI)

S1 Table Identification of new HCV host factors in a functional siRNA screen. This table summarises the data plotted in Fig 1a and 1b. Gene IDs are from the NCBI RefSeq database. Genes are ordered according to their mean score for HCV assembly and release. Significant effects on HCV early or late replication stages are shown in bold characters (same significance criteria as in Fig 1). Controls are in italics. The p-value significance thresholds are the same as in the rest of the manuscript: ** highly significant (p-value < 0.01), * significant (p-value < 0.05), n.s. non-significant (p-value ≥ 0.1).
(DOC)
