# Supplementary figures and images for: ABHD5/CGI-58, the Chanarin-Dorfman Syndrome Protein, Mobilises Lipid Stores for Hepatitis C Virus Production
Source: PLoS Pathog. 2016 Apr 28;12(4):e1005568. doi: 10.1371/journal.ppat.1005568 (PMC4849665; doi:10.1371/journal.ppat.1005568)

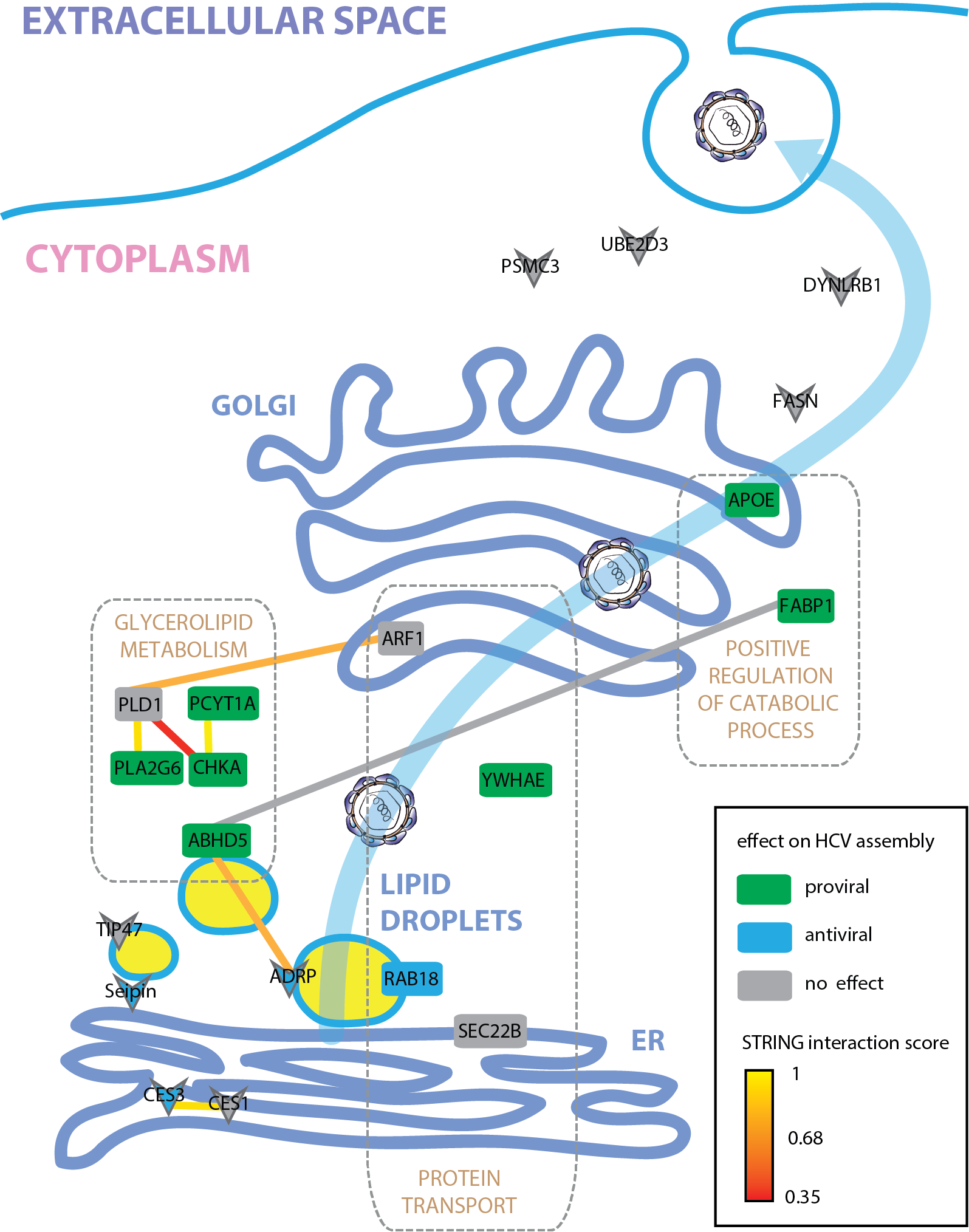

Supplement: S1 Fig — Functional annotation clusters (dotted boxes) and interactions (bold lines) are depicted and proteins are placed in their predominant cellular location. Proteins with proviral and antiviral function on HCV assembly and release are highlighted in green and blue, respectively. Note that a new interaction between ABHD5 and FABP1 was reported [42] but not inventoried in the STRING database yet. For this reason, this interaction was added as a grey line and was not taken into account in the functional clustering. (TIF) [file ppat.1005568.s001.tif]

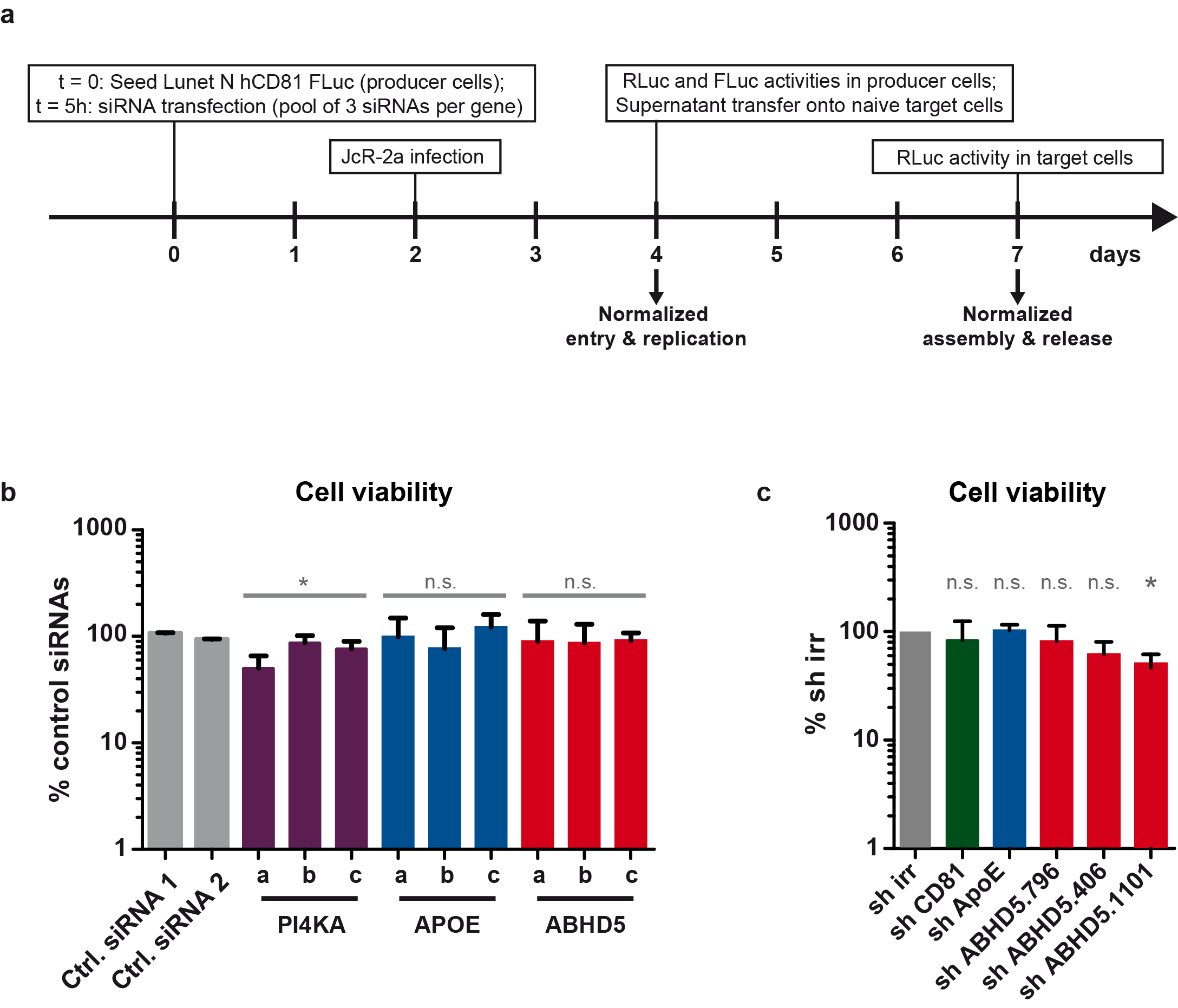

Supplement: S2 Fig — (a) The screen was conducted in Lunet N hCD81-Fluc cells, whose constitutive Firefly luciferase expression was used as a marker for cell viability. The cells were transfected with a pool of 3 siRNAs against each target gene and infected with a Renilla luciferase-reporter virus (JcR-2a). The RLuc activity in the “producer” cell lysates, once corrected for the cell viability is an indicator of HCV entry and RNA replication (see results in Fig 1a). Finally, naive Lunet N hCD81-Fluc cells were infected with the supernatant of the siRNA-transfected and HCV-infected producer cells. The RLuc activity in these “target” cells therefore reflects, once corrected for HCV entry and RNA replication, the efficiency of HCV production (see results in Fig 1b). (b, c) Effect of the ABHD5-specific siRNAs (panel b, data relating to Fig 1c and 1d) or shRNAs (panel c, data relating to Fig 1e, 1f and 1g) on the cell viability. Cell viability was determined by the FLuc activity in the producer cell lysates at the time of virus harvest. (TIF) [file ppat.1005568.s002.tif]

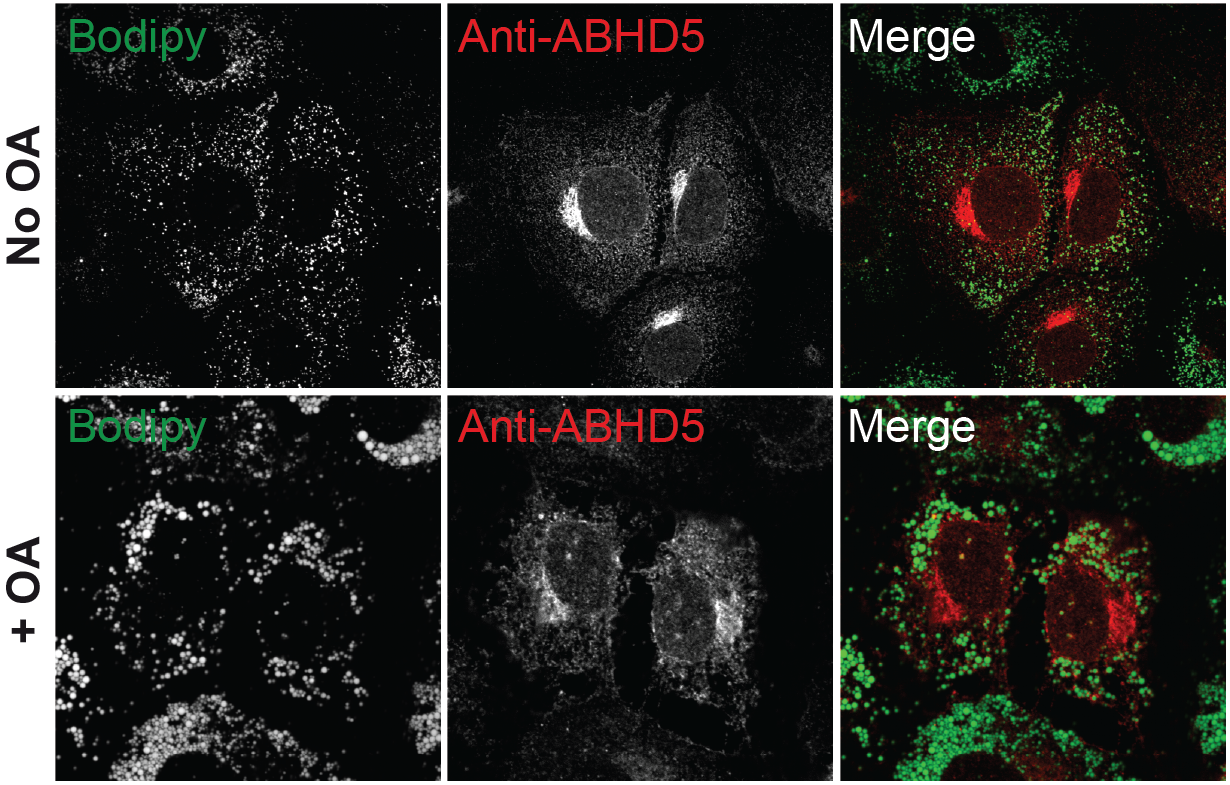

Supplement: S3 Fig — Untagged ABHD5 was expressed by lentiviral transduction in the Lunet N hCD81 cell line. The cells were fixed 48 h post-transduction, after, when applicable, overnight induction with oleic acid (bottom row). Samples were stained with anti-ABHD5 antibody and with Bodipy. (TIF) [file ppat.1005568.s003.tif]

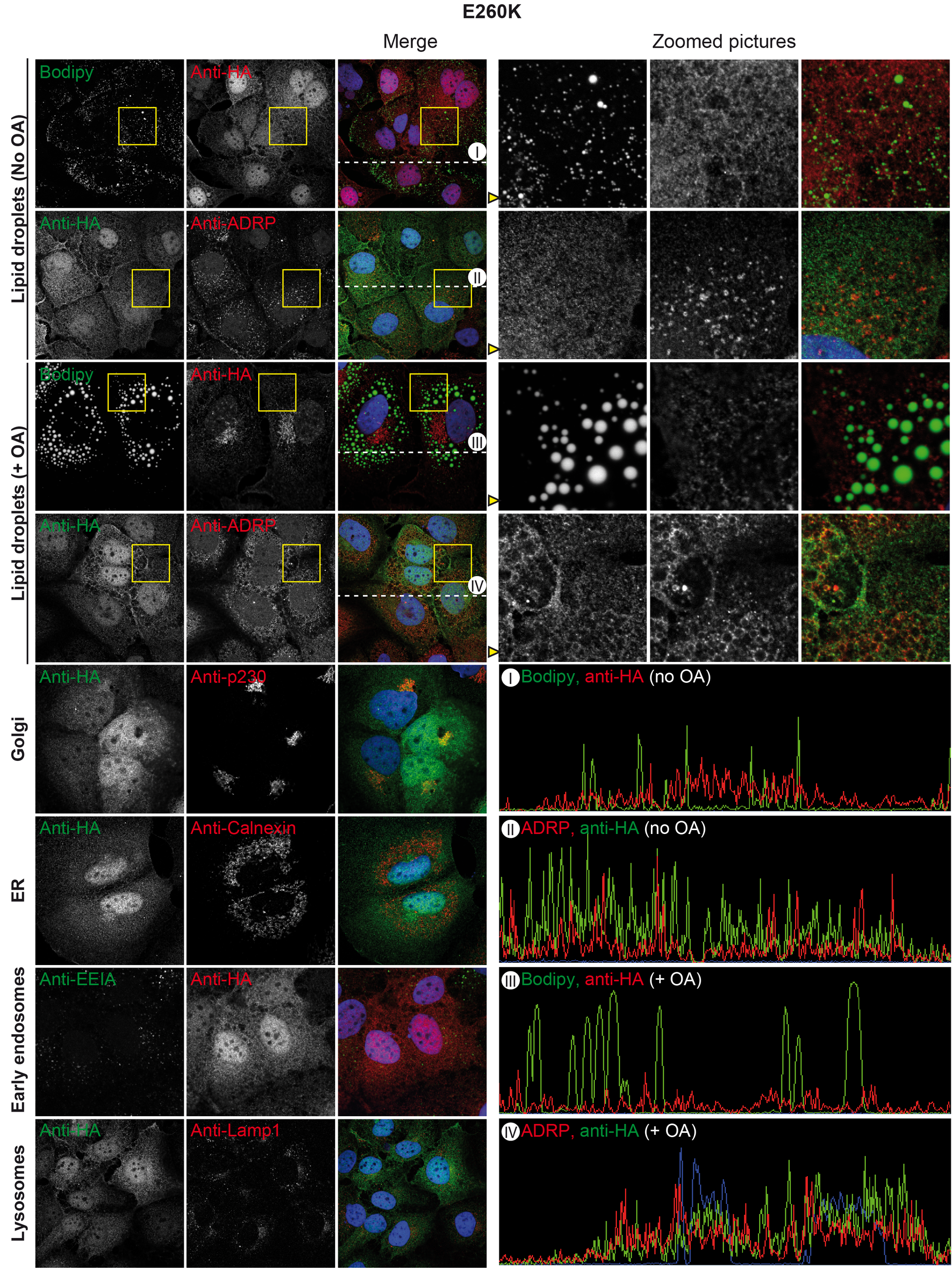

Supplement: S4 Fig — The localisation of the E260K mutant was analysed the same way as for the wild-type and Q130P variant in Figs 3 and 4, respectively. This figure displays representative pictures while the phenotypes quantified over 2 independent experiments are depicted in Fig 5. (TIF) [file ppat.1005568.s004.tif]

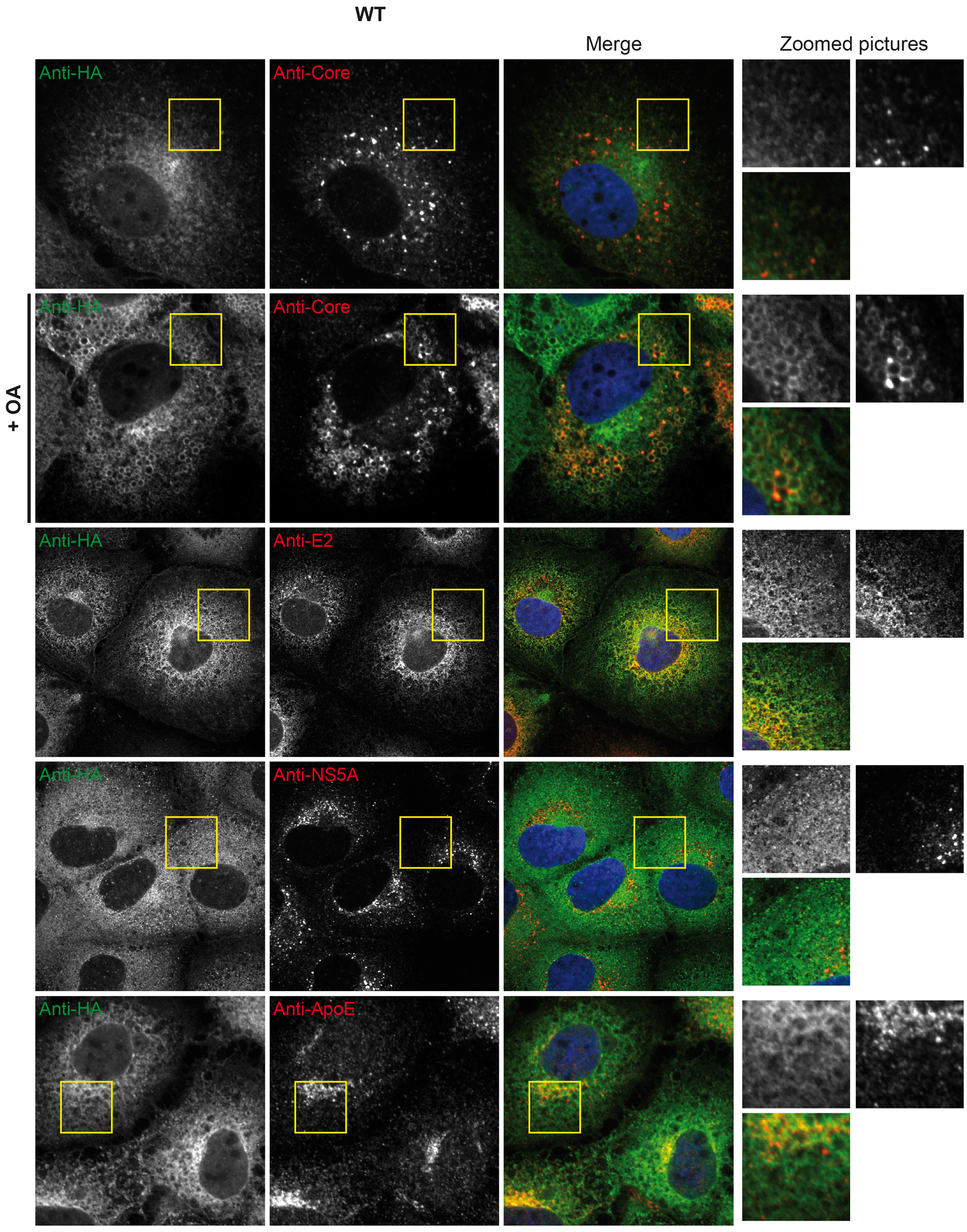

Supplement: S5 Fig — Lunet N hCD81 cells were infected with Jc1 virus and transduced to express HA-tagged wild-type ABHD5. Cells were stained for the HA epitope as well as for diverse HCV proteins or ApoE. For each picture, a portion of the image highlighted with a yellow square is magnified on the right side and depicted in the different channels in the same order. (TIF) [file ppat.1005568.s005.tif]

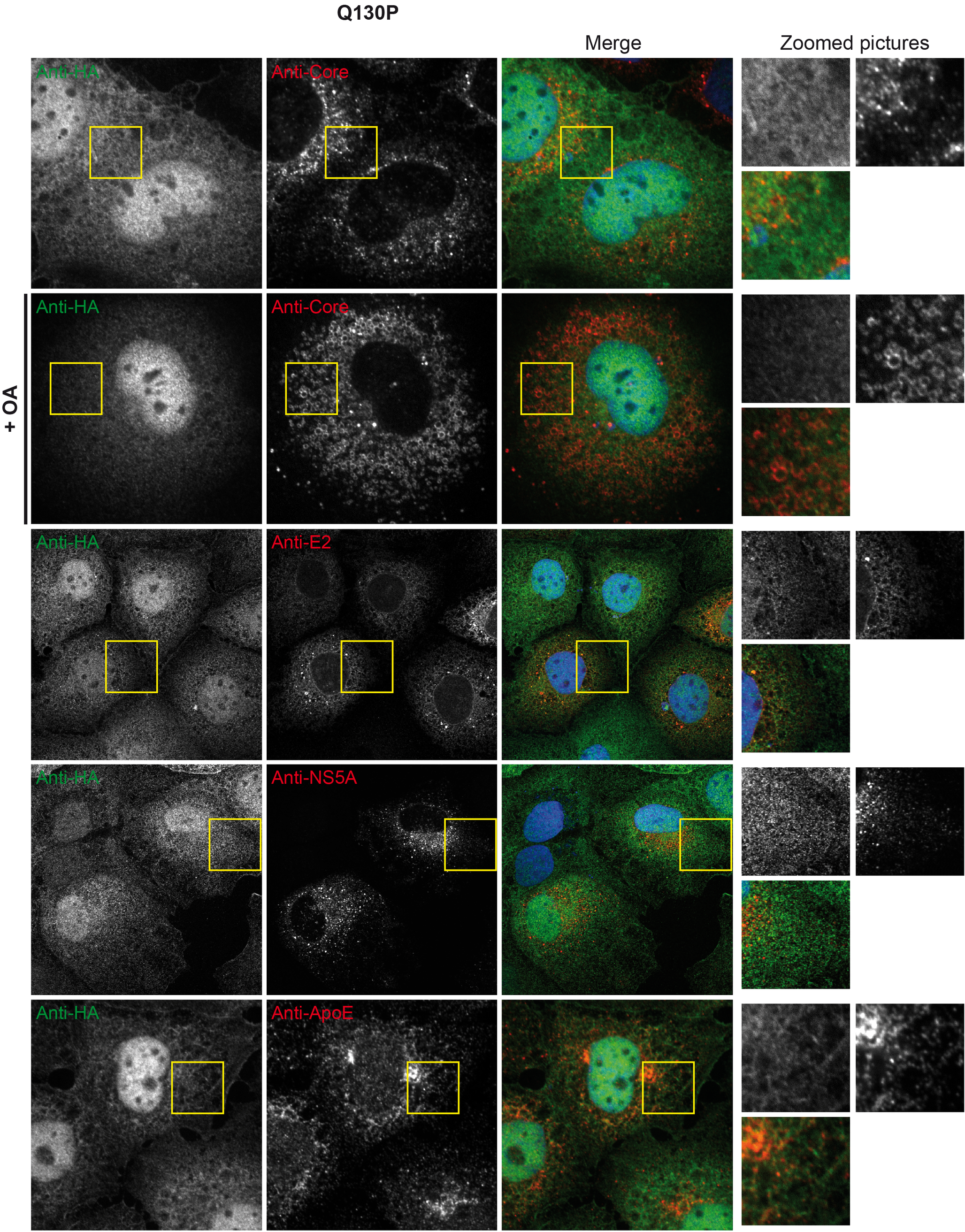

Supplement: S6 Fig — Lunet N hCD81 cells were infected with Jc1 virus and transduced to express HA-tagged Q130P mutant. Cells were stained and images presented as in S4 Fig. (TIF) [file ppat.1005568.s006.tif]

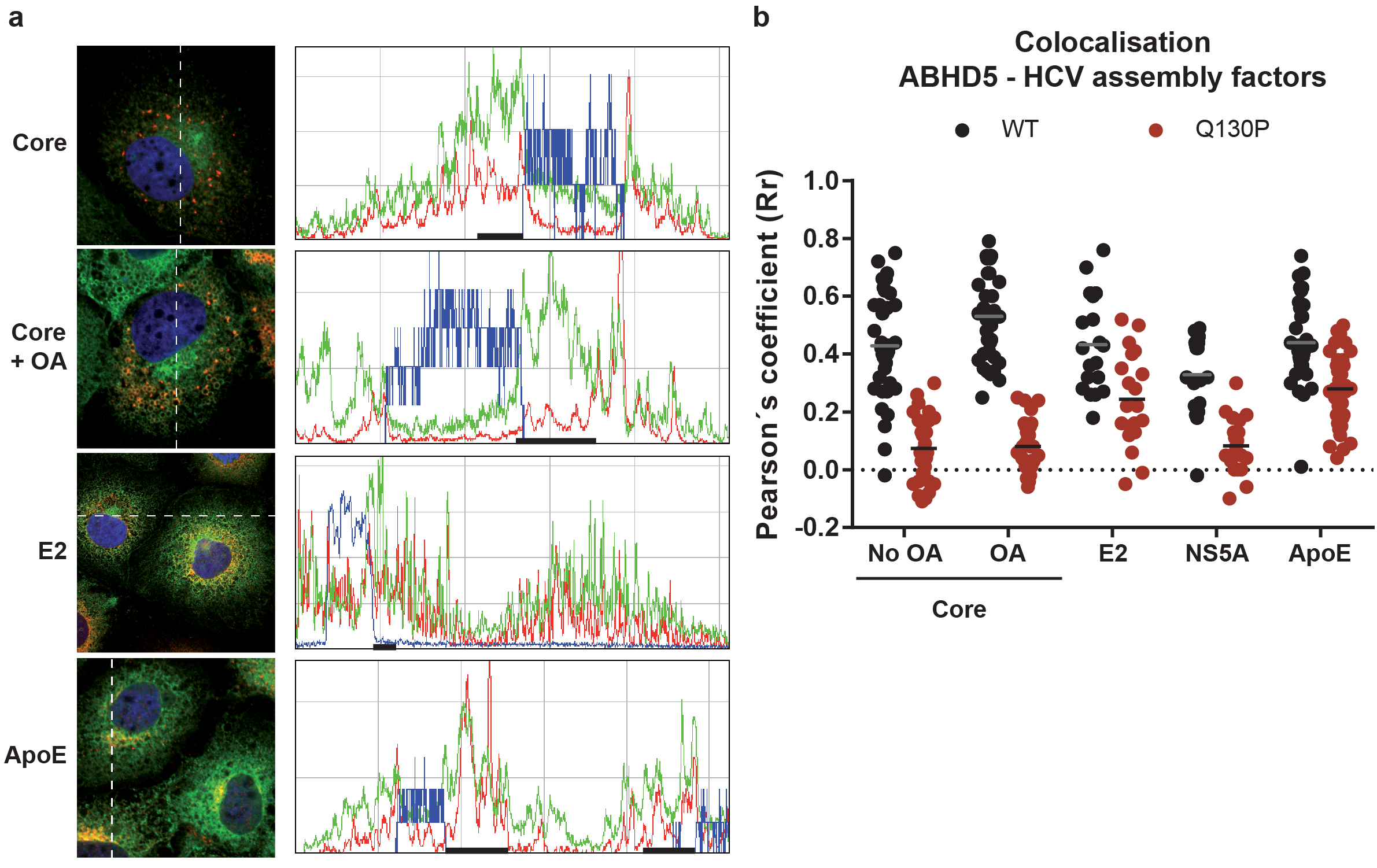

Supplement: S7 Fig — (a) Intensity profiles of wild-type HA-tagged ABHD5 (green), Dapi (blue) and core, E2 or ApoE signals (red) across a section of the images depicted in panel a (see white dotted line). The black rectangles at the bottom of the profiles indicate the approximate position of the Golgi apparatus, as suggested by the concentration of the ABHD5 staining. (b) Colocalisation between HA-tagged ABHD5 and HCV proteins or ApoE was assessed with the Pearson’s correlation coefficient (Rr) calculated over 2 (E2, NS5A) to 3 (core, ApoE) independent experiments and 9–15 frames per experiment. Note that for each frame, Rr was calculated over a ROI corresponding to the double-positive cells (transduced and infected cells). Each dot corresponds to one frame. (TIF) [file ppat.1005568.s007.tif]

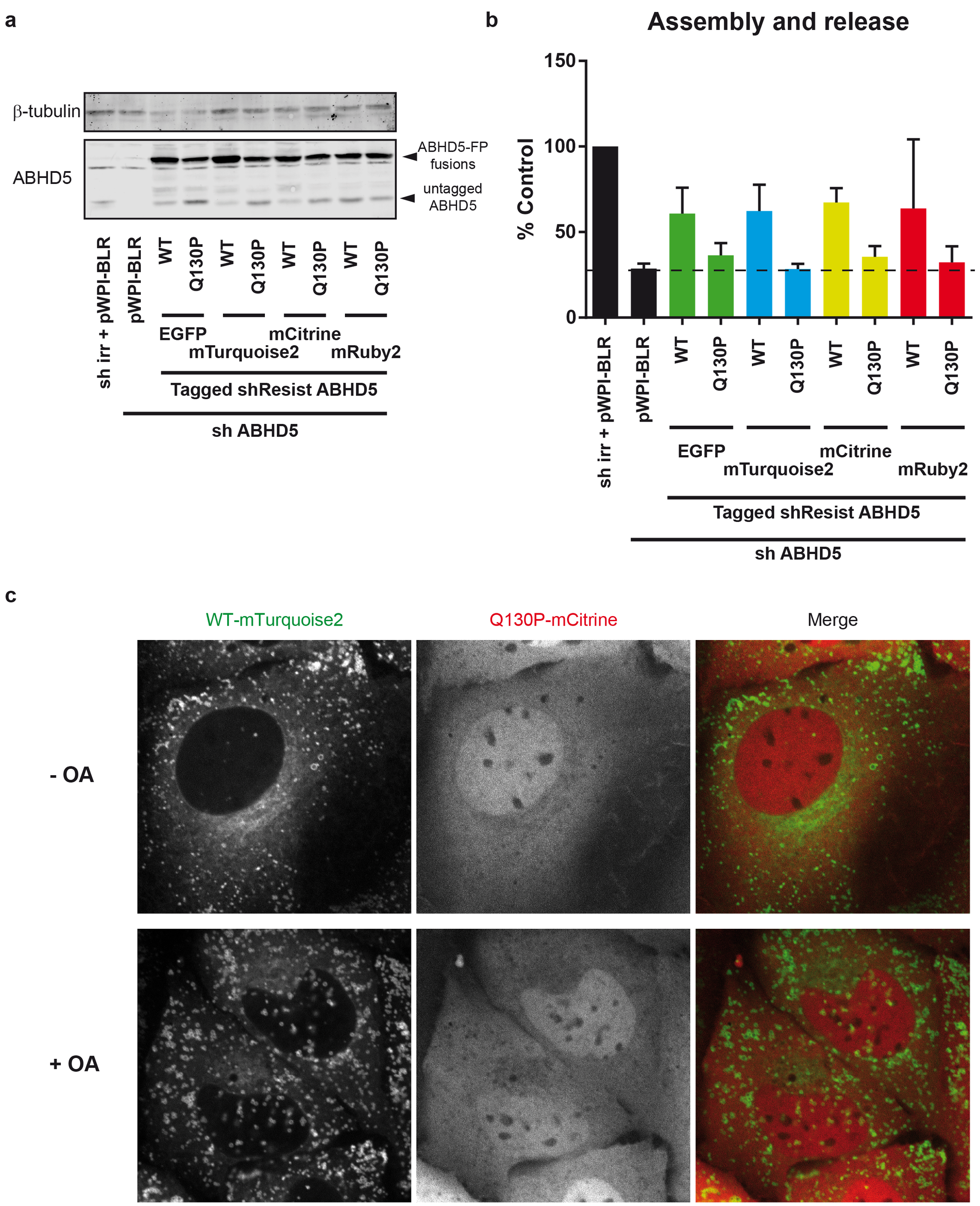

Supplement: S8 Fig — (a, b) Rescue experiment. (a) Western Blot analysis of the expression of the fluorescently tagged ABHD5 constructs 5 days post-transduction (end of HCV infection). Detection of β-tubulin served as an internal control for protein load. (b) Fluorescently tagged ABHD5 constructs support HCV assembly and release. Progeny virion production was analysed by normalising the released infectious titre by the replication values. (c, d) Lunet N hCD81 cells were transduced simultaneously for expression of wild-type and mutant ABHD5. Note that the wild-type construct was fused to mTurquoise2, while the Q130P mutant was fused to mCitrine. Localisation of the two fusion proteins was investigated in untreated (c) or oleic-acid-treated cells (d). Note that WT-mTurquoise2 and Q130P-mCitrine are shown in green and red, respectively. For 3 dimensional reconstitutions, please see S1 and S2 Videos. (TIF) [file ppat.1005568.s008.tif]

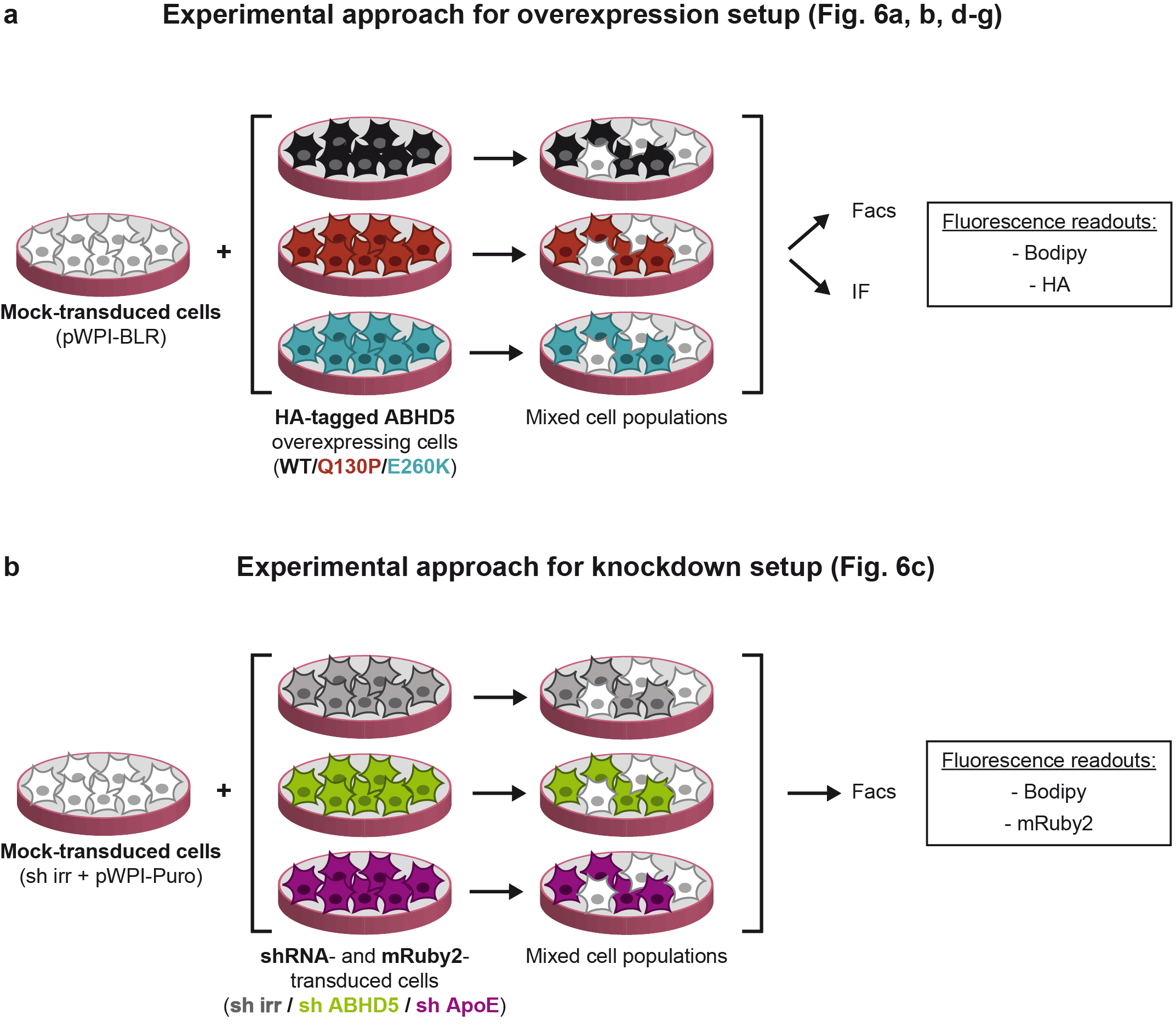

Supplement: S9 Fig — This figure summarises the approach used in Fig 6, with the overexpression (a) and knockdown (b) setups. Please see the legend of Fig 6 for a description of the experiment. (TIF) [file ppat.1005568.s009.tif]

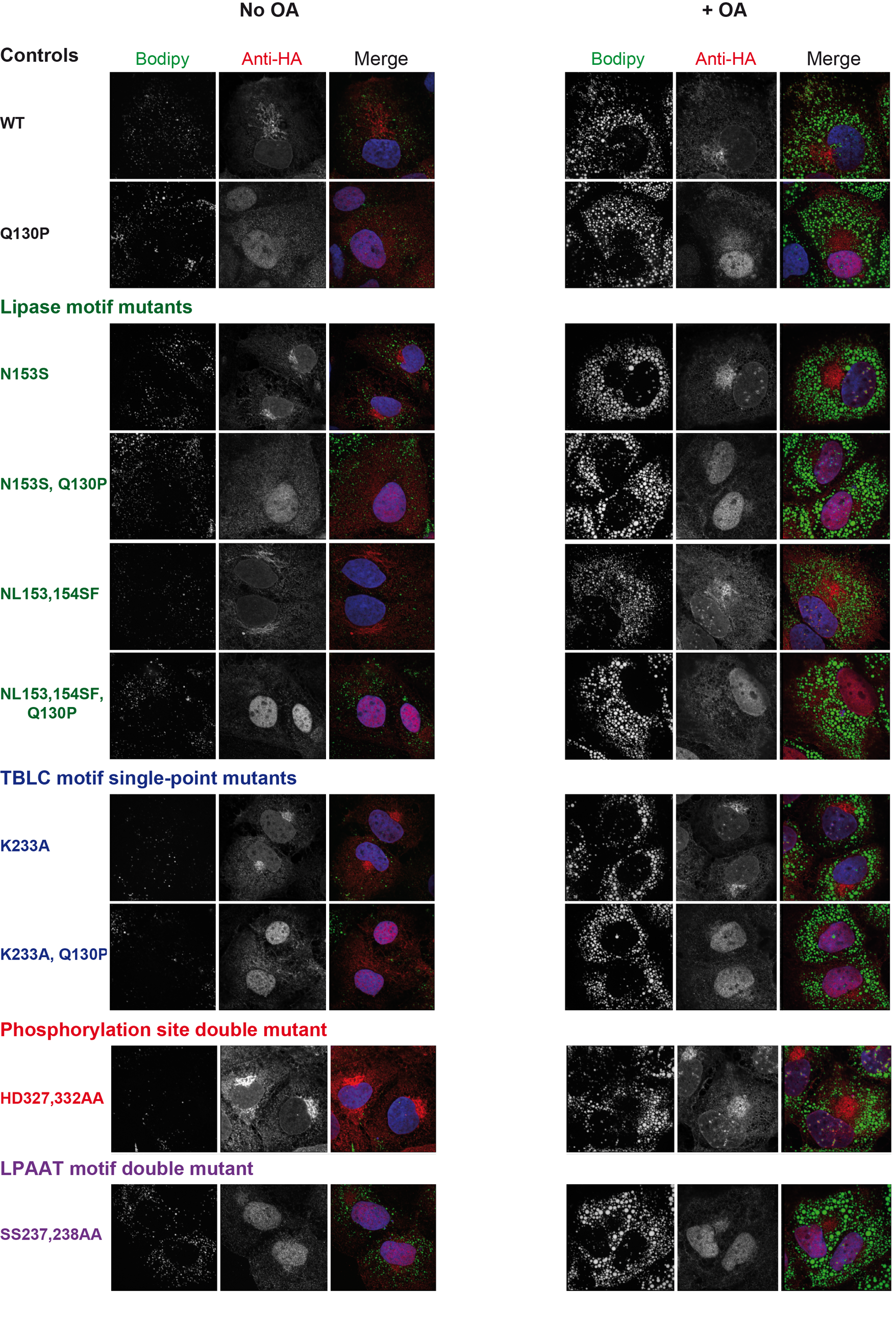

Supplement: S10 Fig — Note that together with S11 Fig, this figure reproduces and extends the data presented in Fig 7e to the complete list of mutants introduced in Fig 7. In this figure, the mutants corresponding to Fig 7a–7d, left panels, are shown. Representative pictures illustrating the subcellular localisation of the mutants, see the legend of Fig 7e for a detailed description. (TIF) [file ppat.1005568.s010.tif]

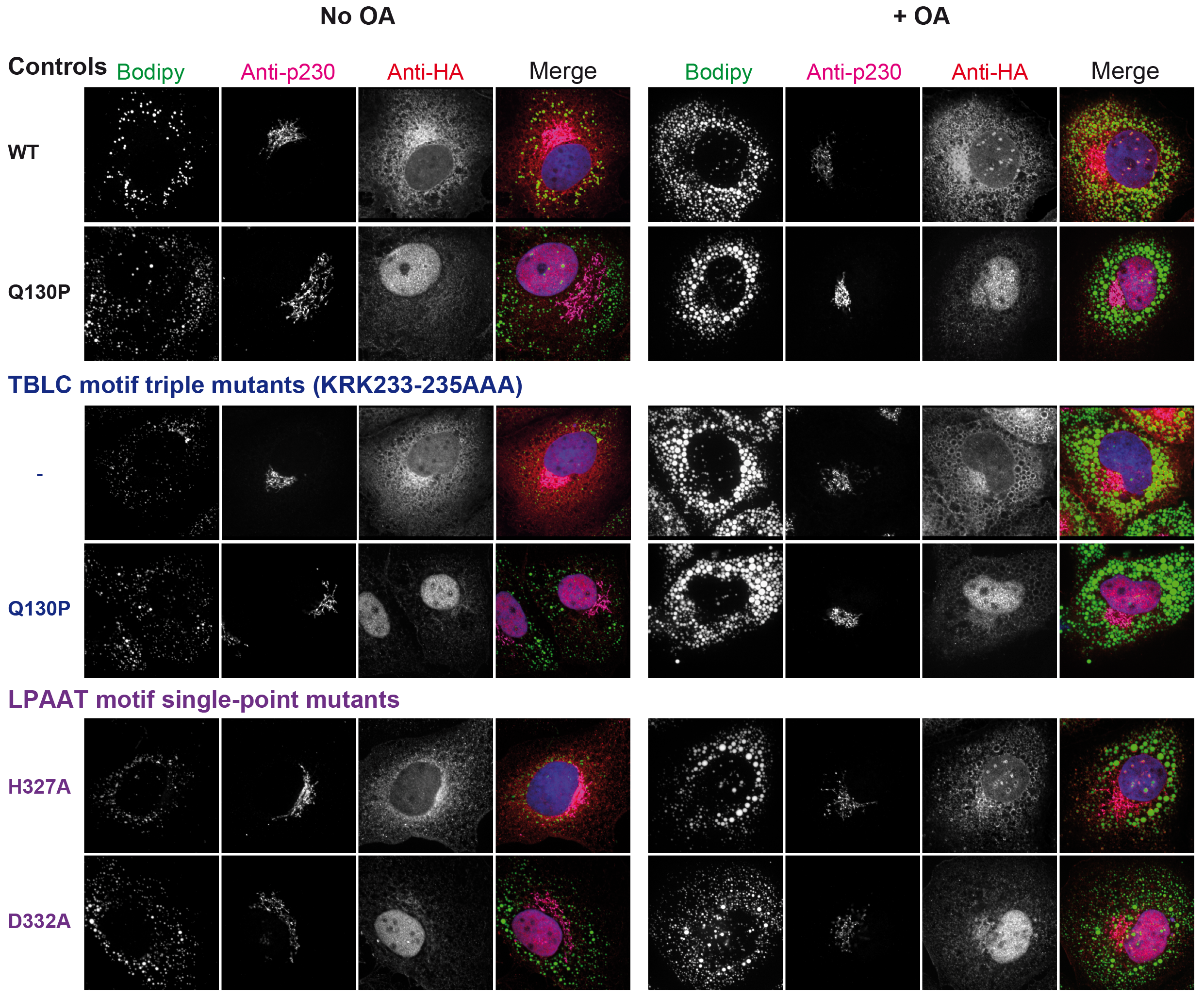

Supplement: S11 Fig — Note that together with S10 Fig, this figure reproduces and extends the data presented in Fig 7e to the complete list of mutants introduced in Fig 7. In this figure, the mutants corresponding to Fig 7a–7d, right panels, are shown. Representative pictures illustrating the subcellular localisation of the mutants, see the legend of Fig 7e for a detailed description. (TIF) [file ppat.1005568.s011.tif]

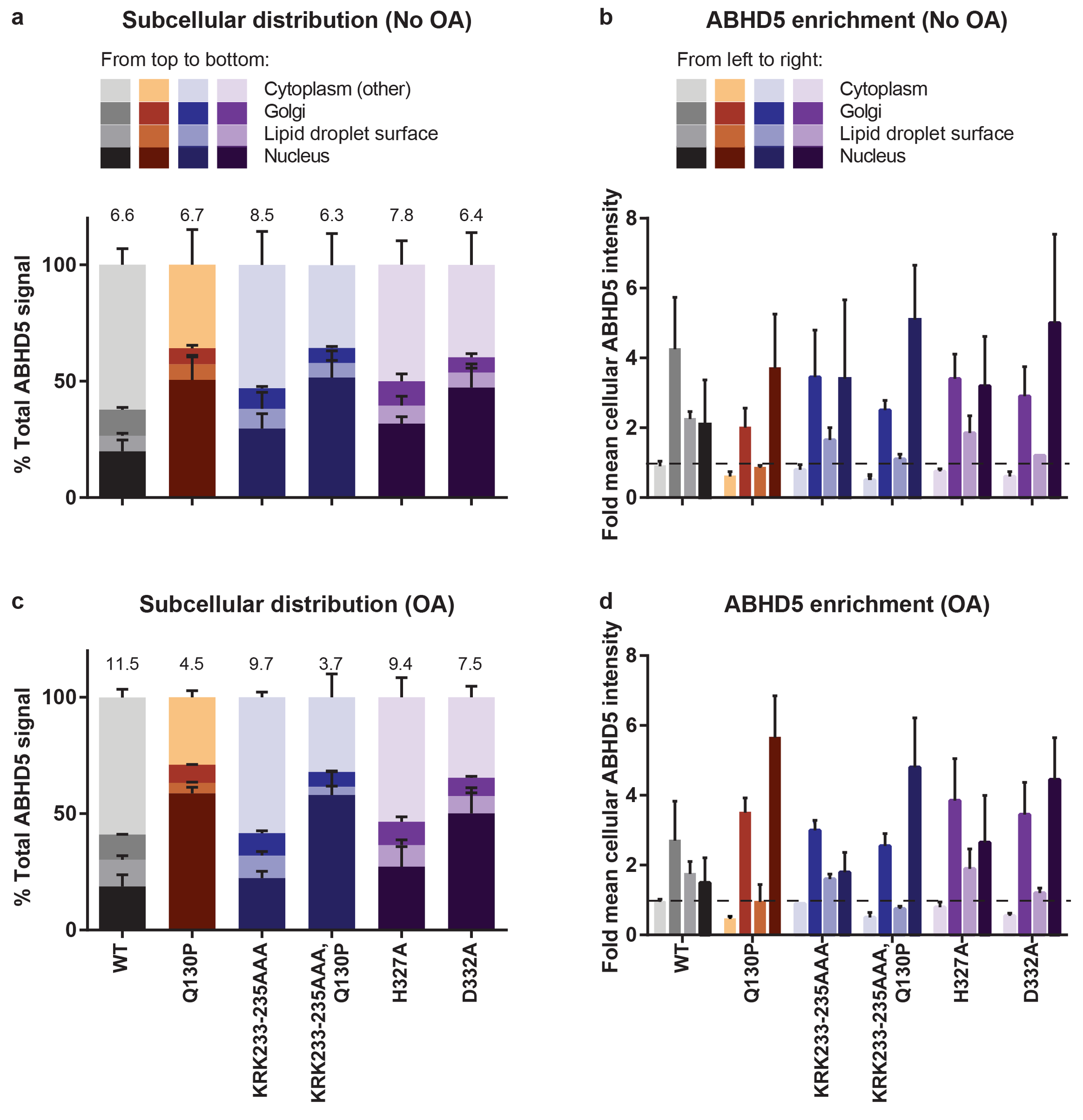

Supplement: S12 Fig — The subcellular distribution (a, b) and enrichment (c, d) of ABHD5 was analysed as in Fig 5 for some of the mutants depicted in Fig 7, in absence (a, c) or presence (b, d) of oleic acid. The numbers on top of each bar correspond to the percentage of LD-associated ABHD5. The data correspond to the average of 2 independent experiments with 10 frames per experiment and construct and representative pictures are to be found in S11 Fig. (TIF) [file ppat.1005568.s012.tif]
