# Supplementary material for: ABHD5/CGI-58, the Chanarin-Dorfman Syndrome Protein, Mobilises Lipid Stores for Hepatitis C Virus Production
Source: PLoS Pathog. 2016 Apr 28;12(4):e1005568. doi: 10.1371/journal.ppat.1005568 (PMC4849665; doi:10.1371/journal.ppat.1005568)
Supplement: S1 Table — This table summarises the data plotted in Fig 1a and 1b. Gene IDs are from the NCBI RefSeq database. Genes are ordered according to their mean score for HCV assembly and release. Significant effects on HCV early or late replication stages are shown in bold characters (same significance criteria as in Fig 1). Controls are in italics. The p-value significance thresholds are the same as in the rest of the manuscript: ** highly significant (p-value < 0.01), * significant (p-value < 0.05), n.s. non-significant (p-value ≥ 0.1). (DOC) [file ppat.1005568.s015.doc]

| **Gene symbol** | **Gene ID** | **Normalised entry and replication** | | | **Normalised assembly and release** | | |
| --- | --- | --- | --- | --- | --- | --- | --- |
|  |  | **Median Score** | **p-value** | **Significance** | **Median Score** | **p-value** | **Significance** |
| PLIN2 / ADRP | 123 | -1,72 | 0,1376 | n.s. | **9,43** | **0,0274** | ***** |
| CES3 | 23491 | -1,62 | 0,1182 | n.s. | **4,22** | **0,0057** | ****** |
| BSCL2 / Seipin | 26580 | -1,68 | 0,0153 | * | **3,25** | **0,0448** | ***** |
| PSMC3 | 5702 | **-4,34** | **0,0129** | ***** | 2,76 | 0,9043 | n.s. |
| RAB18 | 22931 | -1,07 | 0,4790 | n.s. | **2,61** | **0,0371** | ***** |
| CES1 | 1066 | -0,55 | 0,9311 | n.s. | 1,39 | 0,0708 | n.s. |
| *CD81* | *975* | ***-3,49*** | ***0,0007*** | ******** | *1,10* | *0,0805* | *n.s.* |
| UBE2D3 | 7323 | -1,35 | 0,0217 | * | 0,65 | 0,5734 | n.s. |
| *Ctrl. siRNA 1* |  | *-0,70* | *0,0917* | *n.s.* | *0,64* | *0,1816* | *n.s.* |
| FASN | 2194 | **-6,47** | **0,0004** | ****** | 0,19 | 0,1105 | n.s. |
| DYNLRB1 | 83658 | -1,11 | 0,0065 | ** | -0,59 | 0,5319 | n.s. |
| PLIN3 / TIP47 | 10226 | -1,02 | 0,0309 | * | -0,81 | 0,1117 | n.s. |
| *Ctrl. siRNA 2* |  | *0,34* | *0,2708* | *n.s.* | *-0,87* | *0,0817* | *n.s.* |
| PLD1 | 5337 | **-2,59** | **0,0102** | ***** | -1,48 | 0,0403 | * |
| FABP1 | 2168 | -1,44 | 0,2850 | n.s. | **-2,56** | **0,0001** | ****** |
| YWHAE | 7531 | **-2,90** | **0,0046** | ****** | **-2,71** | **0,0267** | ***** |
| SEC22B | 9554 | **-2,01** | **0,0131** | ***** | -3,57 | 0,0613 | n.s. |
| ARF1 | 375 | **-4,48** | **0,0022** | ****** | -4,14 | 0,0958 | n.s. |
| *PI4KA* | *5297* | ***-12,61*** | ***0,0038*** | ******** | *-7,16* | *0,0555* | *n.s.* |
| ABHD5 | 51099 | -1,88 | 0,0019 | ** | **-7,87** | **0,0064** | ****** |
| *APOE* | *348* | ***-2,02*** | ***0,0047*** | ******** | ***-10,43*** | ***0,0054*** | ******** |
| CHKA | 1119 | **-7,06** | **0,0049** | ****** | **-11,08** | **0,0042** | ****** |
| PCYT1A | 5130 | **-3,35** | **0,0006** | ****** | **-11,27** | **0,0126** | ***** |
| PLA2G6 | 8398 | -1,30 | 0,0096 | ** | **-14,11** | **0,0028** | ****** |
